# A novel insight into nitrogen and auxin signaling in lateral root formation in tea plant [*Camellia sinensis* (L.) O. Kuntze]

**DOI:** 10.1186/s12870-020-02448-7

**Published:** 2020-05-24

**Authors:** Shunkai Hu, Mi Zhang, Yiqing Yang, Wei Xuan, Zhongwei Zou, Emmanuel Arkorful, Yi Chen, Qingping Ma, Anburaj Jeyaraj, Xuan Chen, Xinghui Li

**Affiliations:** 1 College of Horticulture, Nanjing Agricultural University, Nanjing, 210095 China; 2 College of Resources and Environmental Sciences, Nanjing Agricultural University, Nanjing, 210095 China; 3 Department of Plant Science, University of Manitoba, Winnipeg, R3T 2N2 Canada

**Keywords:** Nitrogen, Auxin, Lateral roots, Signaling pathway, *Camellia sinensis*

## Abstract

**Background:**

Tea plant (*Camellia sinensis*) is one of the most popular non-alcoholic beverages worldwide. In tea, lateral roots (LRs) are the main organ responsible for the absorption of moisture and mineral nutrients from the soil. Lateral roots formation and development are regulated by the nitrogen and auxin signaling pathways. In order to understand the role of auxin and nitrogen signaling in LRs formation and development, transcriptome analysis was employed to investigate the differentially expressed genes involved in lateral roots of tea plants treated with indole-3-butyric acid (IBA), *N-1-naphthylphthalamic* acid (NPA), low and high concentrations of nitrogen.

**Results:**

A total of 296 common differentially expressed genes were identified and annotated to four signaling pathways, including nitrogen metabolism, plant hormone signal transduction, glutathione metabolism and transcription factors. RNA-sequencing results revealed that majority of differentially expressed genes play important roles in nitrogen metabolism and hormonal signal transduction. Low nitrogen condition induced the biosynthesis of auxin and accumulation of transcripts, thereby, regulating lateral roots formation. Furthermore, metabolism of cytokinin and ethylene biosynthesis were also involved in lateral roots development. Transcription factors like *MYB* genes also contributed to lateral roots formation of tea plants through secondary cell wall biosynthesis. Reversed phase ultra performance liquid chromatography (RP-UPLC) results showed that the auxin concentration increased with the decreased nitrogen level in lateral roots. Thus, tea plant lateral roots formation could be induced by low nitrogen concentration via auxin biosynthesis and accumulation.

**Conclusion:**

This study provided insights into the mechanisms associated with nitrogen and auxin signaling pathways in LRs formation and provides information on the efficient utilization of nitrogen in tea plant at the genetic level.

## Background

Plant’s ability to explore the soil environments for water and nutrients is highly dependent on the architecture of its root systems [1, 2]. The lateral roots (LRs) have high physiological activities, and allow plants to adapt to various nutrients, temperatures and soil conditions [3–5].

Lateral roots formation is affected by several factors, among which, nitrogen and auxin are the prominent factors. Nitrogen is a crucial component for the synthesis of most biological compounds such as DNA, amino acids, proteins and plant hormones [6]. Plant roots can absorb nitrogen in organic (amino acids and peptides) and inorganic (nitrate and ammonium nitrogen) forms [7]. The plasticity of lateral roots is sensitive to different nitrogen suppliers [8, 9]. It has been reported that excess application of nitrate can inhibit LRs development, while lower nitrate level enhances the growth of LRs [9]. On the contrary, LRs growth can be inhibited at supra-optimal N supply [10, 11]. It is reported that nitrate transporter genes (NRTs) are responsible for the high-affinity NO_3_^−^ transport system, which in turn induce LRs growth [12]. Interestingly, Okamoto et al. (2003) found that the expression levels of *NRT1.1* and *NRT2.1* can be strongly induced by treating plants with high level of NO_3_ [13]. According to Remans et al. (2006), *NRT1.1* can regulate the growth of primary and lateral roots; *NRT2.1* plays a major role in the absorption of NO_3_^−^ in *Arabisopsis*, and also determines the root architecture by controlling LRs formation [14]. The *ANR1* (A NO_3_^−^-inducible Arabidopsis gene) was the first identified gene involved in signaling transduction, which is associated with NO_3_ concentration, and consequently affects LRs development [15]. The ammonia transporter gene *AtAmt1.1* has also been found to play a critical role in restructuring LRs architecture under N-starvation [16]. Vidal et al. (2010) identified a passive feedforward mechanism consisting of auxin receptor AUXIN SIGNALING F-Box 3 (AFB3) and microRNA 393 (miRNA 393), which lead to the auxin-NO_3_^−^ pathway [12]. The auxin biosynthesis gene *TAR2* (tryptophan aminotransferase related 2) is also reported to reprogram root architecture under low nitrogen conditions [17]. Therefore, nitrogen supplying has a strong correlation with auxin synthesis and metabolism. These reports have provided a clear basis for further exploration of the regulatory relationship between nitrogen metabolism-related genes and plant LRs growth. However, most of these studies focused on the model plant *Arabidopsis thaliana* only, and rarely included woody plants.

Plant hormonal signals participate in various plant growth and developmental stages, including embryogenesis, seed germination, vegetative growth, fruit ripening and leaf senescence. Therefore, the exploration of plant hormones and their regulatory roles in growth and development of LRs have been extensively reported. Studies have reported that abscisic acid (ABA) restrains LRs development after emergence of LRs primordium from primary root and before excitation of LRs meristem [18]. Auxins regulate LRs formation through the stimulation of several endogenous and environmental signals [19]. Cytokinin also affects primary and LRs initiation, organ differentiation. The role of cytokinin in root formation is a factor of cytokinin-to-auxin ratio. A high cytokinin-to-auxin ratio inhibits root formation and vise versa [20]. Gibberellins in combination with other hormones (ABA, ethylene, and auxin) controls LRs formation [21]. Plant hormonal involvement in LRs formation is controlled by complex signal crosstalk. However, the potential molecular mechanisms of hormonal involvement in LRs formation remain unclear.

Tea plant [*Camellia sinensis* (L.) O. Kuntze] is one of the most popular non-alcoholic natural beverages worldwide. It is a nitrogen-preferring perennial woody plant having well-developed LRs. Nitrogen fertilizer is essential for tea leaf increasing and quality. LRs are the main organs used for tea plant to absorb soil nutrients and water. Therefore, LRs development has a direct impact on yield and quality of tea plant. Developed LRs increase the absorption capacity of tea plant for soil nitrogen. The effects of nitrogen and auxin relationship on LRs formation has not been explored. Owing to this, this study aimed to indentify the differentially expressed genes involved in the formation of LRs in tea plant under nitrogen-deficient, nitrogen-toxicity, IBA (Indole-3-butyric acid, root development enhancement), NPA (*N − 1-naphthylphthalamic* acid, root development inhibition) treatments. The concentration of auxin, mechanisms underlying LRs phenotypic changes and their responses to different nitrogen concentrations in tea plants were also investigated. The study also proposed the signaling pathways and regulatory networks of nitrogen and auxin in LRs.

## Results

### Tea plant growth performance in response to diverse N levels and auxin

**Fig. 1:**
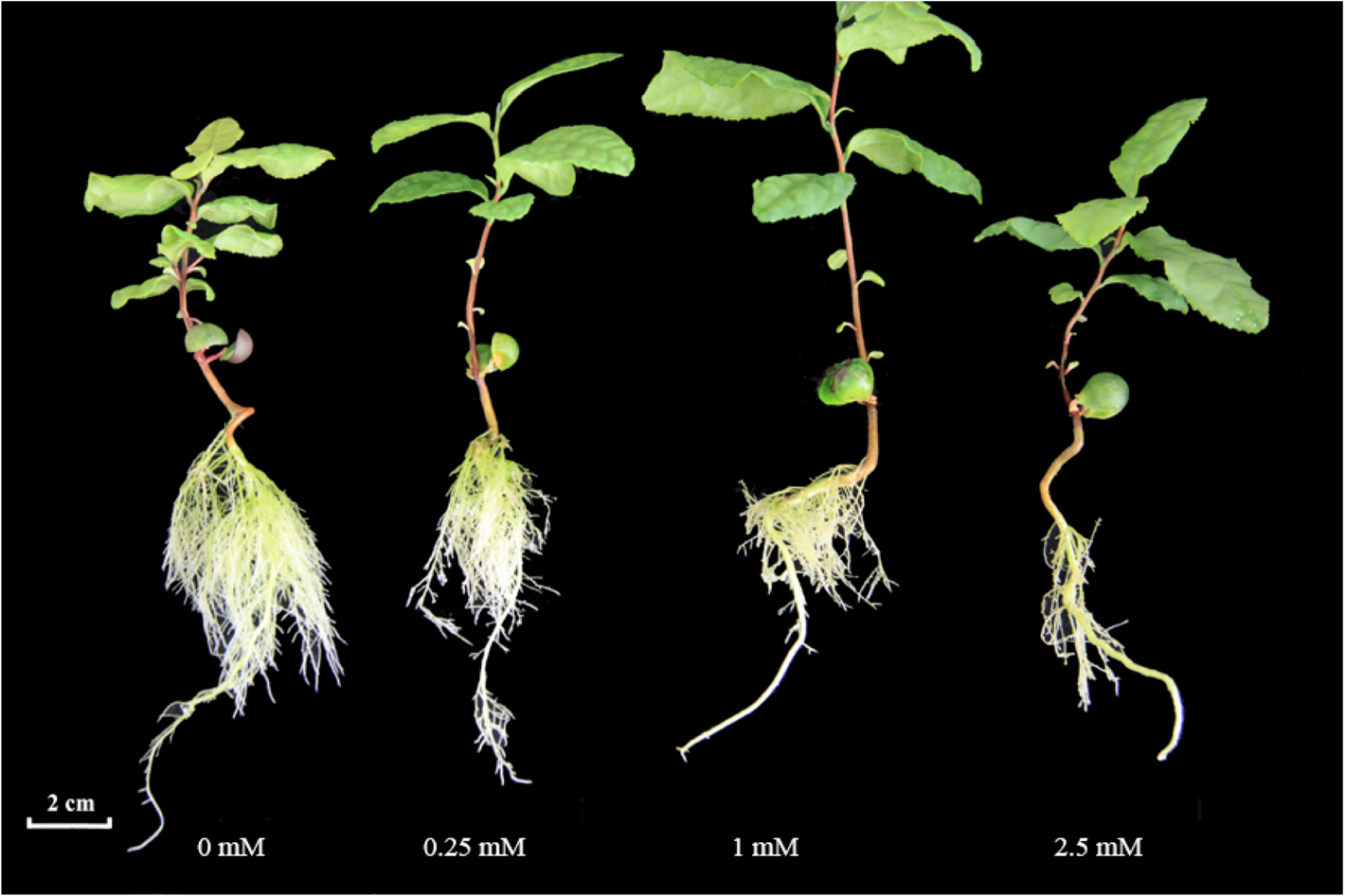
Shoot growth performance of tea plant in hydroponics culture supplemented with 0, 0.25, 1 and 2.5 mM nitrogen for 10 weeks

**Fig. 2:**
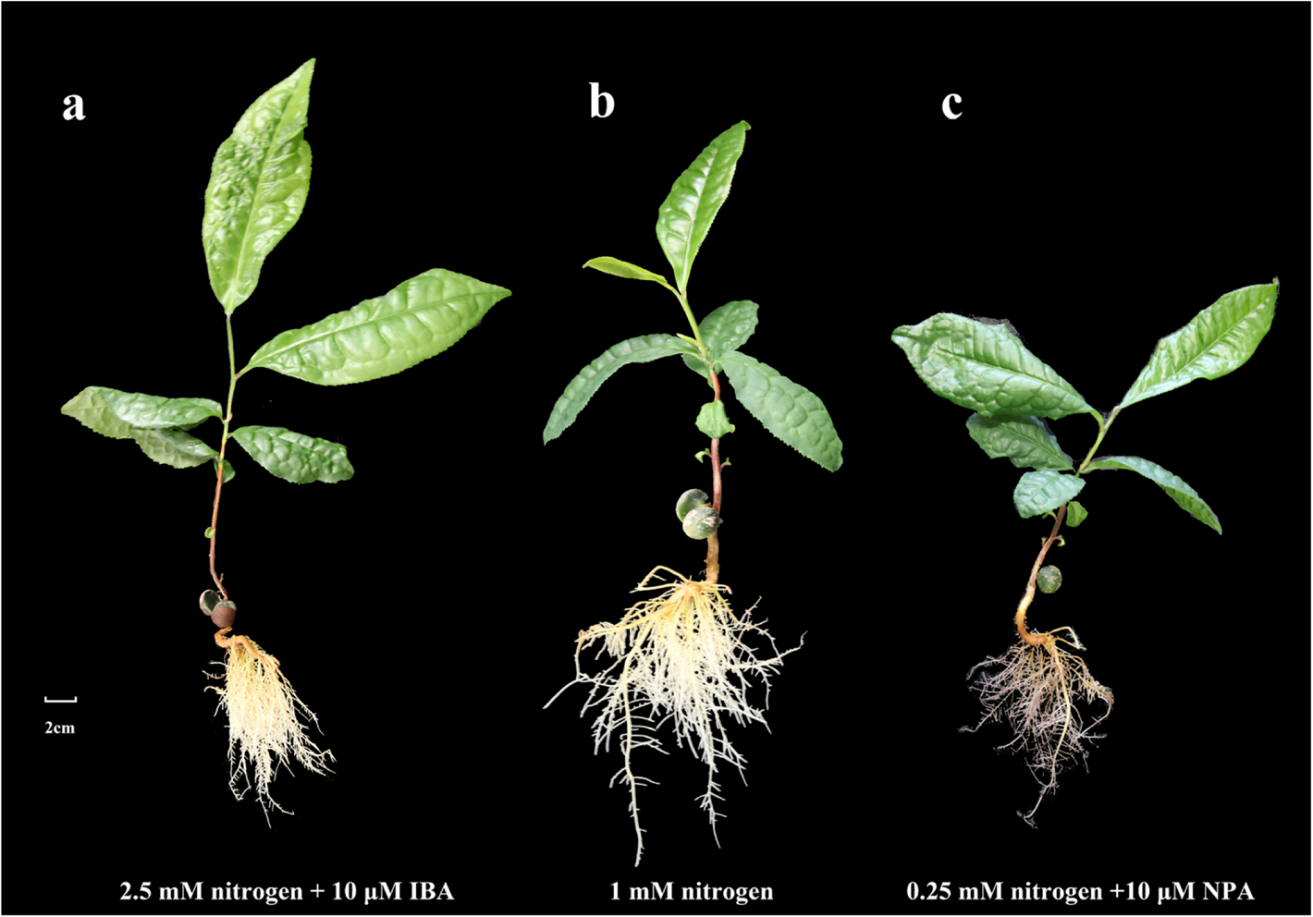
Shoot growth performance of tea plant in hydroponics culture supplemented with (a) 2.5 mM nitrogen for 8 weeks, then with 10 μM IBA for 8 weeks (b) 0.25 mM nitrogen for 8 weeks, then with 10 μM NPA for 4 weeks

**Fig. 3:**
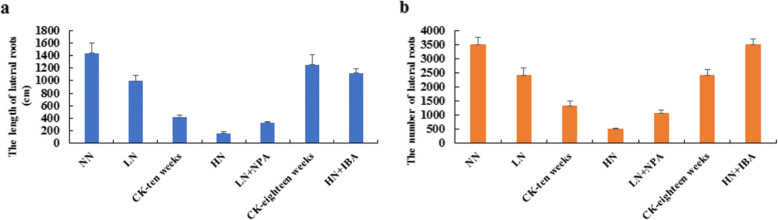
**a** The length of lateral roots in hydroponics culture. **b** The number of lateral roots in hydroponics culture

The tea seedlings showed normal leaves growth under various nitrogen treatments in hydroponic culture. All seedlings showed varied condition of growth after 10 weeks, resulting in significant change with N treatment (Fig. 1). Primary roots of seedlings were thinner and longer with lower nitrogen treatment. The seedlings showed greener leaves, shortened length of lateral roots, and significant reduction in numbers of LRs as N concentration was increased. The aboveground parts of seedlings treated with NN (no nitrogen), LN (low nitrogen) and HN (high nitrogen) were shorter than the control (Fig. 1). However, as nitrogen concentration increased, the number and length of lateral roots decreased significantly (Fig. 3). These results indicate that low nitrogen concentration promotes tea LRs formation and development while high nitrogen concentration inhibits LRs growth. In addition, when the seedlings were treated with hormones, growth of LRs was significantly affected. The results showed that HN + IBA treatment (2.5 mM nitrogen for 10 weeks, and then cultured with 2.5 mM nitrogen + 10 μM IBA for 8 weeks) restored seedlings’ LRs development, and the number and length of LRs were increased after 8 weeks (Fig. 2a, Fig. 3). Compared with LN treatment, LN + NPA treatment (0.25 mM nitrogen for 10 weeks, and then cultured with 0.25 mM nitrogen + 10 μM NPA for 4 weeks) inhibited and obviously decreased seedlings’ LRs development. The leaves of the seedlings also showed brownish colouration (Fig. 2c, Fig. 3). This suggests that exogenous auxin could avoid the inhibition of the LRs growth with HN treatment in tea plant. Therefore, since auxin accumulation and production were inhibited by HN treatment, LRs growth would be restrained by exogenous auxin (IBA).

### Effects of nitrogen and IBA supply on auxin and zeatin concentrations in LRs

**Fig. 4:**
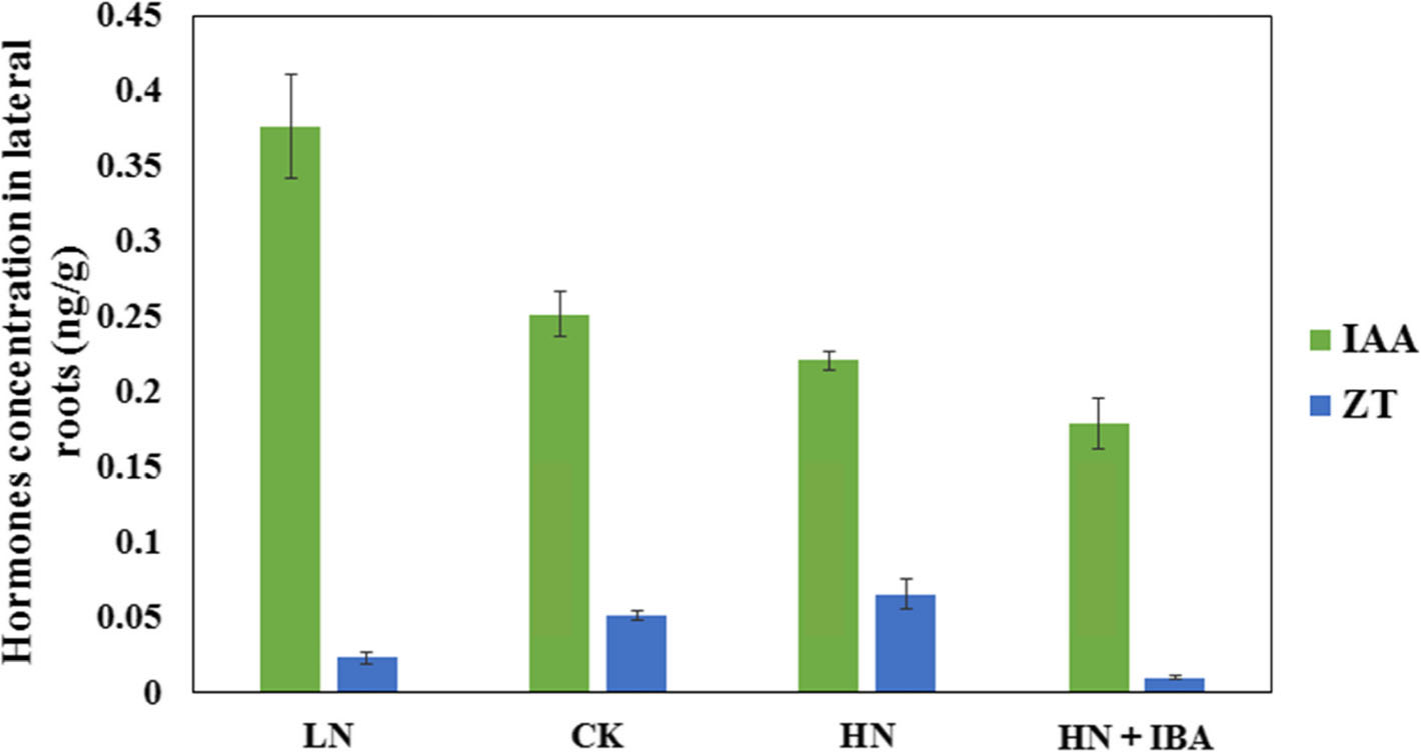
Effects of nitrogen and auxin treatments on indole-3-acetic acid (IAA) and zeatin (ZT) concentrations in lateral roots

There was a significant reduction of auxin concentration in LRs treated with HN concentration (Fig. 4). This suggests that HN could inhibit production of auxin, while low nitrogen could induce production of auxin in LRs. It also showed that concentration of auxin in LRs decreases when exogenous auxin concentration increases. There was a positive correlation between the zeatin concentration and nitrogen concentration of LRs. Therefore, zeatin concentration in LRs was also increased when seedlings were treated with HN concentration. Also, high exogenous auxin concentration decreased zeatin concentration in LRs.

### Transcriptomes data analysis

**Fig. 5:**
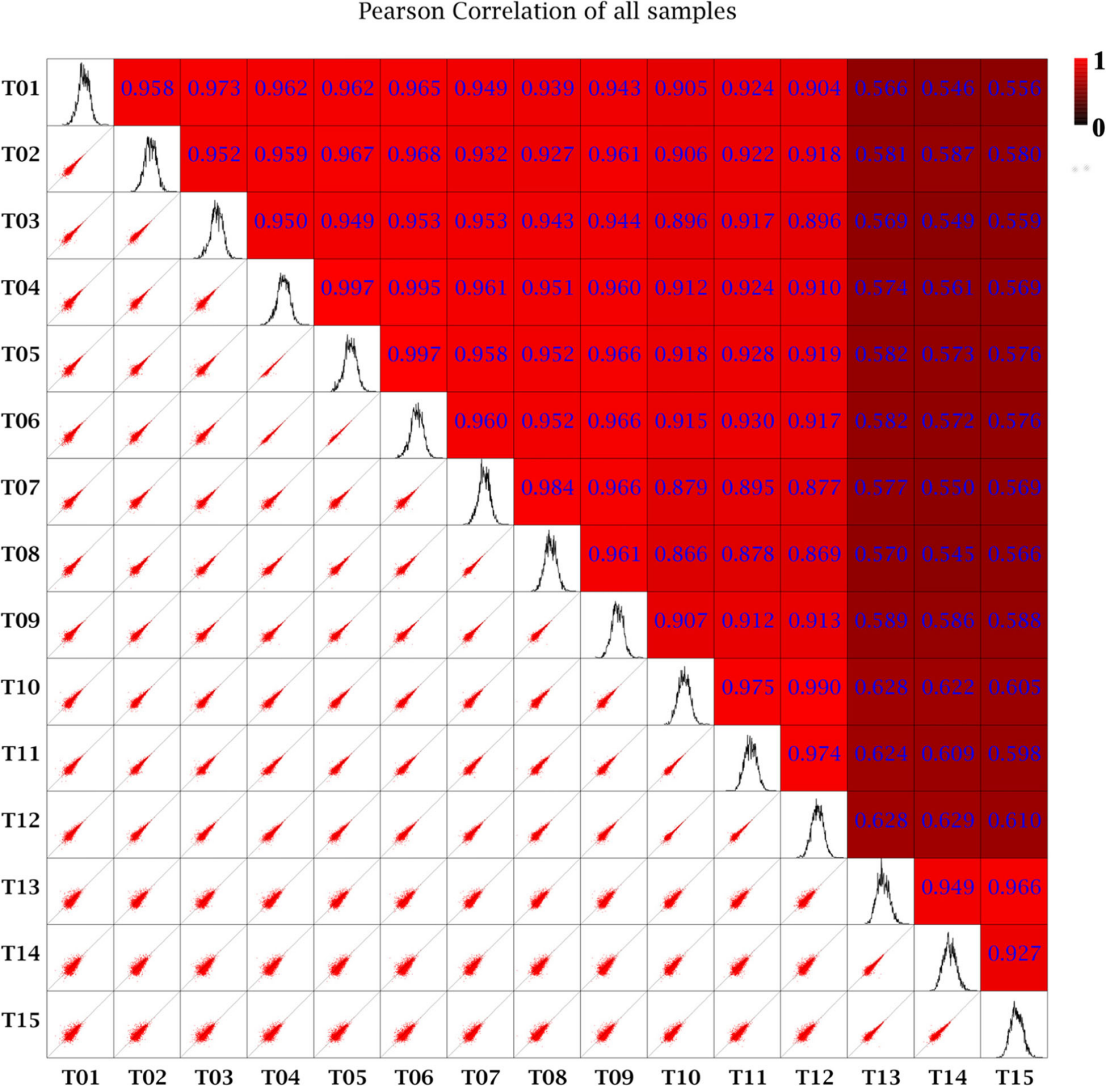
Pearson correlation analysis of CK, LN, HN, LN + NPA and HN + IBA treatments on lateral roots of *Camellia sinensis*. T1, T2, T3 are analysis of LN; T4, T5, T6 are analysis of CK; T7, T8, T9 are analysis of HN; T10, T11, T12 are analysis of LN + NPA; and T13, T14, T15 are analysis of HN + IBA treatments

A total of 136.13 Gb clean data with a minimum Q30 of 92.83% was obtained (Table 1). The clean reads of each sample were sequence-aligned with the designated reference genome, and the efficiency of the alignment was over 76.06%. Sample correlation coefficients of data obtained revealed that the throughput and quality of sequencing were high enough for further analysis (Fig. 5).

**Table 1:** Quality assessment of clean data

Samples	Read number	Base number	GC content	% ≥ Q30
LN-1	22,300,039	6,690,011,700	45.28	93.81
LN-2	22,352,271	6,705,681,300	45.25	94.08
LN-3	71,345,574	21,403,672,200	45.24	92.83
LN + NPA-1	22,625,999	6,787,799,700	45.23	94.01
LN + NPA-2	22,937,652	6,881,295,600	45.69	94.27
LN + NPA-3	20,972,742	6,291,822,600	45.10	93.45
N-CK-1	50,566,454	15,169,936,200	44.84	93.86
N-CK-2	26,485,890	7,945,767,000	44.82	94.03
N-CK −3	51,958,794	15,587,638,200	44.90	94.57
HN + IBA-1	24,283,790	7,285,137,000	45.18	93.86
HN + IBA-2	25,455,012	7,636,503,600	45.26	94.41
HN + IBA-3	24,543,852	7,363,155,600	44.89	94.12
HN-1	22,116,811	6,635,043,300	44.92	93.97
HN-2	23,245,627	6,973,688,100	44.83	93.64
HN-3	22,586,710	6,776,013,000	44.74	93.67

LN (0.25 mM nitrogen for 10 weeks + 24 h), CK (the control, 1 mM nitrogen for 10 weeks + 24 h), HN (2.5 mM nitrogen for 10 weeks + 24 h), LN + NPA (0.25 mM nitrogen for 10 weeks, and then cultured with 0.25 mM nitrogen + 10 μM NPA for 24 h), HN + IBA (2.5 mM nitrogen for 10 weeks, and then cultured with 2.5 mM nitrogen + 10 μM IBA for 24 h)

### Function annotation of the tea LRs transcriptome

**Fig. 6:**
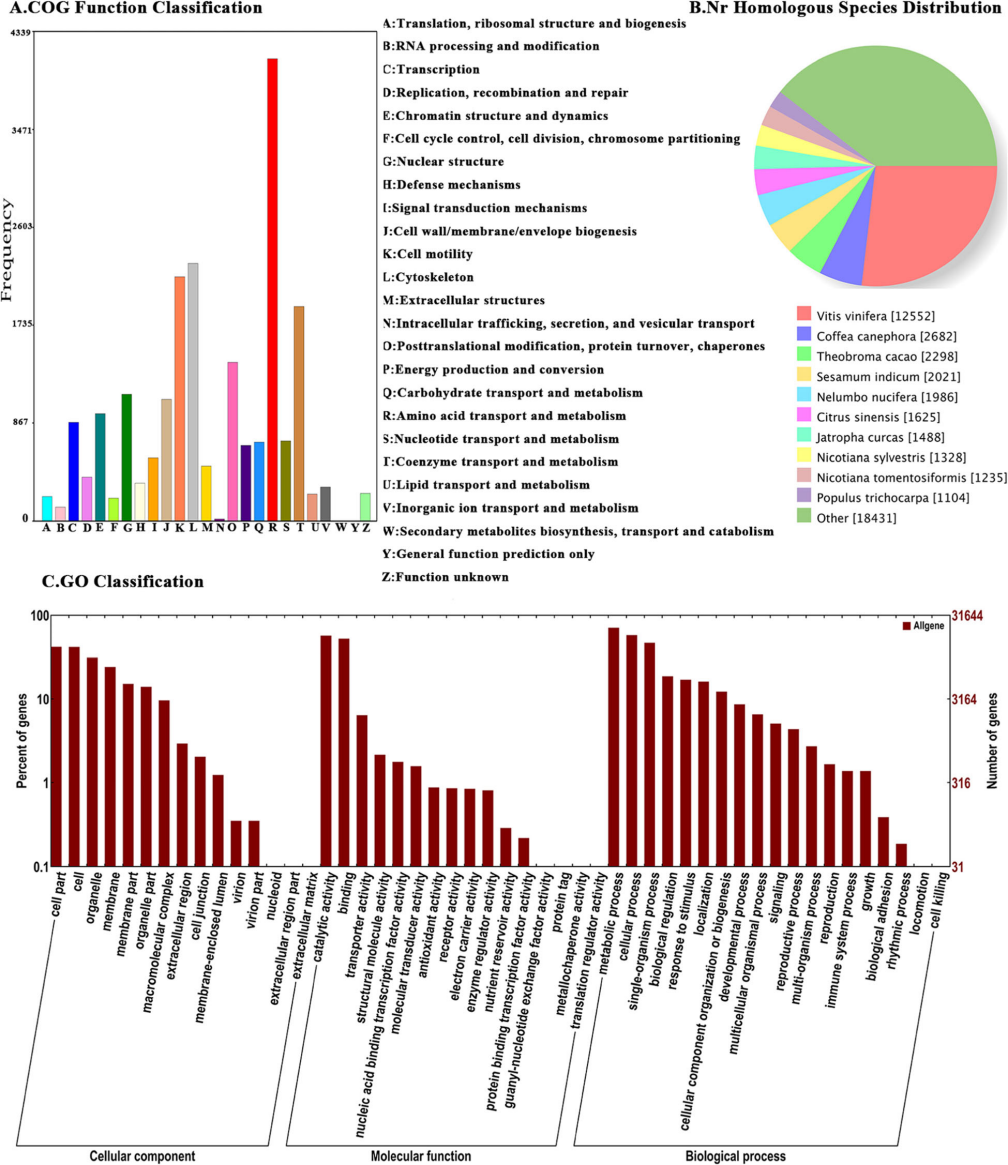
Functional annotation and classification of all unigenes identified in tea plant, as determined by Cluster of Orthologous Groups (COG), Non-redundant (NR) and gene ontology (GO) databases

Assembled unigenes were annotated with NR, NT, Swiss-Port, KEGG, COG and GO databases. Annotation to NR database revealed that 12,552 NR annotated unigenes showed high identity to *Vitis vinifera*, which was more similar than other species (Fig. 6). The assembled genes associated with ‘metabolic process’, which the ‘single-organism process’ and ‘cellular process’ represented the majority of GO classification. In cellular component, a total of 13,249, 13,180, and 9876 unigenes were annotated in cell part, cell and organelle, respectively. In molecular function, a total of 17,987 unigenes were annotated in catalytic activity and 16,499 unigenes were annotated to binding. In biological process, a total of 22,439, 18,248 and 14,766 unigenes were annotated in metabolic process, cellular process and single-organismal process, respectively. COG functional classification analysis also revealed that most unigenes were annotated to ‘amino acid transport and metabolism’, cytoskeleton and cell motility.

### GO and KEGG analysis of differentially expressed genes

**Fig. 7:**
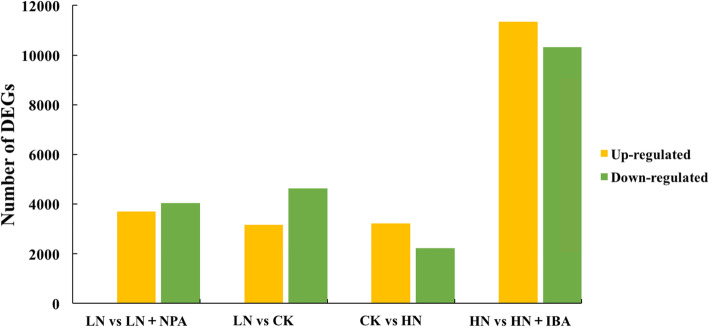
Number of DEGs between LN vs LN + NPA, LN vs CK, CK vs HN, HN vs HN + IBA treatments

A total of 7784 DEGs were identified from the LRs of seedlings treated with LN and CK, and among them, a total of 3164 and 4622 unigenes were up-regulated and down-regulated, respectively. A total of 5432 unigenes (3216 up-regulated and 2219 down-regulated unigenes) were differentially expressed in LRs of seedlings treated with HN. Also, a total of 7756 DEGs (3709 up-regulated and 4047 down-regulated unigenes) were obtained from the LRs of seedlings treated with LN in the presence or absence of NPA. There were 21,671 DEGs in the LRs between the seedlings treated with HN in the presence or absence of IBA, of which 11,338 and 10,333 were up-regulated and down-regulated, respectively (Table 2, Fig. 7).

**Table 2:** Corresponding annotated DEG number

DEG Set	Total	Swiss-Prot	GO	KEGG	COG	KOG	Pfam	NR
LN vs LN + NPA	4498	3248	2814	449	1304	2402	2728	4462
LN vs CK	4183	2977	2610	387	1108	2261	2427	4136
CK vs HN	2992	2107	1822	279	804	1558	1701	2956
HN vs HN + IBA	15,274	11,300	10,235	1606	5037	8349	10,328	15,209

LN (0.25 mM nitrogen for 10 weeks + 24 h), CK (the control, 1 mM nitrogen for 10 weeks + 24 h), HN (2.5 mM nitrogen for 10 weeks + 24 h), LN + NPA (0.25 mM nitrogen for 10 weeks, and then cultured with 0.25 mM nitrogen + 10 μM NPA for 24 h), HN + IBA (2.5 mM nitrogen for 10 weeks, and then cultured with 2.5 mM nitrogen + 10 μM IBA for 24 h)

Based on functional annotations, the identified DEGs between the two libraries (LN and CK, HN and CK, LN + NPA and LN, HN and HN + IBA) were classified into three Gene Ontology (GO) categories: biological process, molecular function and cellular component. GO cellular component analysis indicated that DEGs between both libraries were involved in cell, cell part and organelle. Molecular function analysis revealed that most DEGs identified were involved in catalytic activity, binding and transporter activity. The identified DEGs contributed to metabolic process, cellular process, and single-organism process (Fig. S1-S4).

Among the DEGs identified from LRs of seedlings between LN and CK treatments, 387 DEGs were assigned to 72 KEGG pathways. Of these pathways, DEGs involved in carbohydrate metabolism (76 genes) were the most abundant, followed by translation (65 genes), and ‘folding, shorting and degradation’ (42 genes) (Fig. S5). Among the DEGs identified from LRs of seedlings between HN and CK treatments, 279 DEGs were assigned to 101 KEGG pathways. Of these, DEGs involved in carbohydrate metabolism (62 genes) were the most abundant, followed by translation (39 genes), and ‘folding, shorting and degradation’ (30 genes) (Fig. S6). In addition, a total of 499 DEGs were assigned to 116 KEGG pathways between the LRs of seedlings treated with LN in the presence or absence of NPA. Of these, 101, 60 and 42 DEGs were involved in carbohydrate metabolism ‘metabolism of other amino acids’, and in amino acids metabolism, respectively (Fig. S7). Finally, 1606 DEGs were assigned to 132 KEGG pathways between LRs of seedlings treated with HN in the presence or absence of IBA. The most abundant of these DEGs were involved in carbohydrate metabolism (274 genes), followed by translation (176 genes), amino acid metabolism (176 genes), lipid metabolism (171 genes) and 171 DEGs in ‘folding, sorting and degradation’ (Fig. S8).

### DEGs related to nitrogen and auxin treatments

**Fig. 8:**
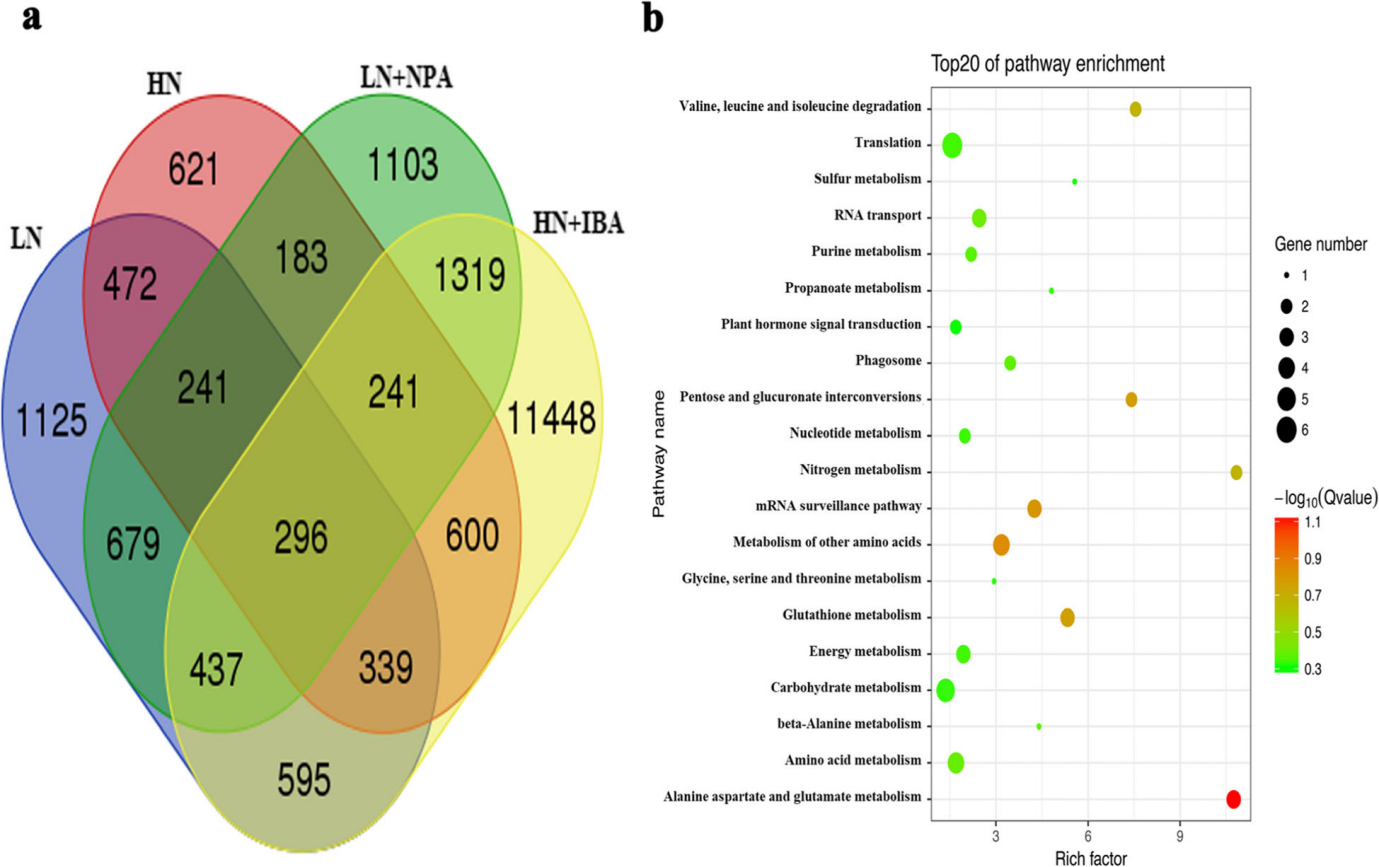
Differentially expressed genes in tea plant lateral roots in response to LN vs CK, CK vs HN, LN vs LN + NPA and HN vs HN + IBA. **a** Venn diagram of common DEGs. **b** Functional distribution of common DEGs in tea plant transcriptome

Finally, a total of 296 common DEGs were identified from the LRs of seedlings in all treatments (Fig. 8a). KEGG enrichment analysis showed that these genes were involved in various metabolic pathways. Most of the DEGs were annotated in nitrogen metabolism, plant hormone signal transduction, glutathione metabolism, and translation (Fig. 8b). In nitrogen metabolism, DEGs included high affinity nitrate transporter (*NRT*) genes, ammonium transporter (*AMT*) gene (Table 3), and nrt1/ptr family (*NPF*) protein genes (Table S2). In the plant hormone signal transduction, the auxin response factor 2 (*ARF2*) genes (Table 3), *Aux/IAA*, *GH3* (indole-3-acetic acid-amido synthetases) genes (Table S2) were identified in auxin metabolism. Adenylate isopentenyltransferase, cytokinin hydroxylases and cytokinin dehydrogenase genes were involved in cytokinin metabolism, while ethylene biosynthetic process genes participate in ethylene metabolism (Table 3, Table S2). In transcription factors, the annotated genes encoding P-type R2R3 MYB protein (Table 3), MADS-box, NAC and WRKY family were identified (Table S2). Glutathione S-transferases (*GSTs*) genes which regulate glutathione metabolism (Table S2), and F-box/kelch genes which participate in F-box protein metabolism were also obtained annotated in treated plants (Table 3).

**Table 3:** DEGs related to nitrogen metabolism, plant hormone signal, transcription factors, Glutathione metabolism and F-box/kelch protein

Gene ID	Log2 (Fold change)				Description
	CK vs LN	CK vs HN	LN vs LN + NPA	HN vs HN + IBA	
CSA011051	1.84	1.53	−3.08	-3.24	High affinity nitrate transporter
MSTRG.51865	1.51	1.23	− 2.73	− 2.72	High affinity nitrate transporter
CSA015778	–	–	–	− 2.56	Tryptophan aminotransferase-related protein 2
CSA018499	−2.32	–	2.61	− 1.71	Ammonium transporter
CSA011327	3.86	–	−4.11	–	Auxin response factor
CSA012843	–	–	–	2.36	Auxin response factor
CSA006753	−1.33	–	–	−3.83	Adenylate isopentenyltransferase
CSA011288	1.16	–	−2.00	–	Cytokinin dehydrogenase
CSA017731	−2.62	–	–	–	Cytokinin hydroxylase
CSA028278	1.16	–	–	–	Ethylene biosynthetic
MSTRG.10434	–	−1.64	–	–	Ethylene biosynthetic
CSA00344	–	–	−1.68	–	Ethylene biosynthetic
CSA017586	–	–	−1.63	–	Ethylene biosynthetic
CSA017852	–	–	–	3.58	Ethylene biosynthetic
CSA003142	–	–	–	6.42	Ethylene biosynthetic
MSTRG.2855	−2.78	–	4.47	−4.25	P-type R2R3 Myb protein
MSTRG.31932	1.71	–	−1.31	–	F-box/kelch protein
CSA036587	–	–	–	1.17	F-box/kelch protein
CSA012447	−1.46	− 1.26	2.74	9.59	Glutathione S-transferase

“–” represents no significant difference in gene expression. LN (0.25 mM nitrogen for 10 weeks + 24 h), CK (the control, 1 mM nitrogen for 10 weeks + 24 h), HN (2.5 mM nitrogen for 10 weeks + 24 h), LN + NPA (0.25 mM nitrogen for 10 weeks, and then cultured with 0.25 mM nitrogen + 10 μM NPA for 24 h), HN + IBA (2.5 mM nitrogen for 10 weeks, and then cultured with 2.5 mM nitrogen + 10 μM IBA for 24 h)

### Relative expression of differentially expressed genes

**Fig. 9:**
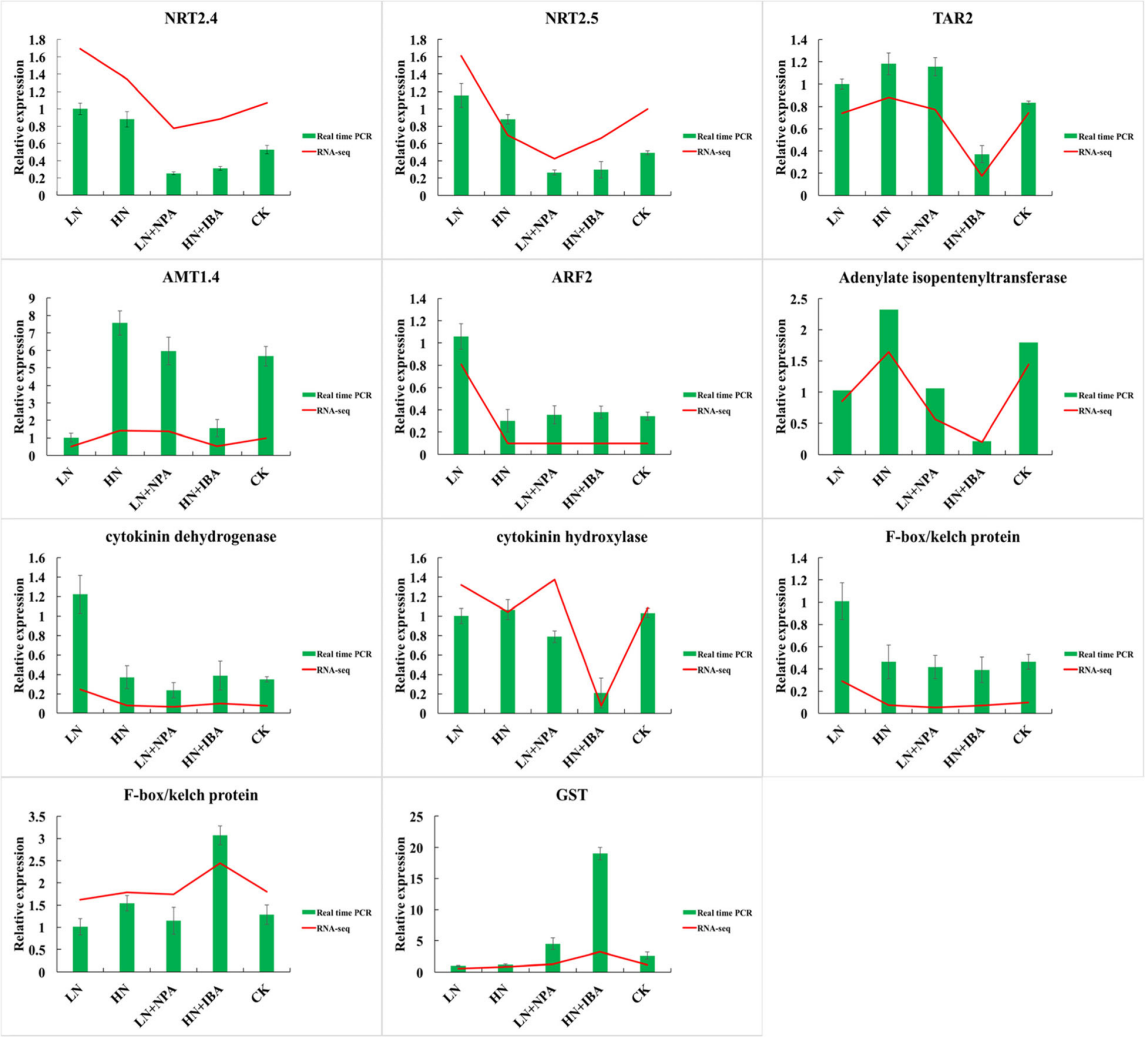
Comparison of expression profiles of selected genes as determined by Real Time-PCR

The reliability of RNA-Seq data was validated by qRT-PCR using eleven genes. The genes are involved in the regulation of nitrogen and auxin signaling pathways. Of these genes, three are associated with nitrogen metabolism; one is associated with tryptophan aminotransferase; one is associated with auxin response factor; two are involved in cytokinin biosynthesis; two are associated with F-box/kelch protein; and one is involved in glutathione metabolism (Table S1). Gene expression analysis revealed that qRT-PCR result was consistent with RNA-Seq analysis, suggesting that RNA-Seq data was reliable (Fig. 9).

## Discussion

### Nitrogen metabolism genes are responsible for various N levels and auxin

Nitrogen and auxin significantly affect LRs development via N signaling, and regulate biosynthesis and transport of hormones such as ABA, GA and IAA. In previous studies, N-deficiency induced high affinity nitrate transporter *NRT1.1*, *NRT2.1*, *NRT2.4* and *NRT2.5* in roots of *Arabidopsis thaliana* plant [22]. It was also reported that *NRT2.1*, *NRT2.2*, *NRT2.4* and *NRT2.5* could synergistically confer to plants the ability to adapt to low N condition [23]. *NRT1* and *NRT2* are not only for NO_3_^−^ transportation but also for auxin transportation under low nitrogen condition. Under the low nitrogen environment, *NRT1* and *NRT2* express in large quantities and then regulate the LRs formation by inducing auxin accumulation and transportation [24]. Similarly, in this study, two common DEGs (CSA011051; MSTRG.51865) *NRT2.4* and *NRT2.5* were identified in LRs under all treatments. *NRT2.4* and *NRT2.5* were up-regulated and down-regulated in the LRs of seedlings under LN and LN + NPA treatments, respectively. In inference, N-deficiency could increase the content of auxin in lateral roots. Therefore, LN treatment could induce *NRT*s expression and thus increase auxin production and accumulation, and could be regulated by nitrogen concentration and auxin treatment, therefore, contributing to tea plant LRs formation.

Auxin and nitrogen signaling could control LR development. Tryptophan (Trp) aminotransferase of Arabidopsis1 (TAA1) is an important enzyme which plays important function in Trp transformation into indole-3-pyruvic acid (IPyA), an auxin biosynthesis pathway (IPyApathway) [25]. Recent studies have shown that TAR regulates plant roots and shoots development: Tryptophan aminotransferase related genes (*TAR1–4*) were involved in IPyA pathway [26], while tryptophan aminotransferase related 2 (*TAR2*) gene was induced by N deficiency that can improve auxin biosynthesis in *Arabidopsis thaliana*, and increase IAA levels in LRs development [27, 28]. *TAR2* is involved in the symthesis and accumulation of auxin in LRs under low nitrogen condition. In the present study, one tryptophan aminotransferase related 2 gene (CSA015778) was down-regulated under HN + IBA treatment, indicating that LN treatment could induce *TAR2* expression for auxin accumulation. However, under excessive exogenous auxin treatment, *TAR2* would be down-regulated to balance LRs auxin concentration. The tryptophan aminotransferase related 4 (*TAR4*) gene was up-regulated under low nitrogen condition in shoots of *Arabidopsis thaliana* [27]. Similarly, *TAR4* (CSA001598) was up-regulated in LRs under LN treatment. This suggests that *TAR4* participate in LRs development under low nitrogen condition. The auxin concentration increased as nitrogen level decreased in LRs of seedlings. In summary, LRs formation in tea plant could be induced by low nitrogen concentration via auxin biosynthesis and accumulation.

*Arabidopsis* has six AMT-type ammonium transporters including *AMT1.1* to *AMT1.5* and *AMT2.1* [29]. Previous reports demonstrated that ammonium supply can induce LRs initiation and branching in *Arabidopsis thaliana*. This could be attributed to the fact that ammonium regulates the development of LRs through a complementary reaction with nitrate, and this reaction occurs in AMT-dependent manners [30]. Ruan et al. (2016) reported that ammonia improves nitrate uptaking in tea roots, however, the present study revealed that *AMT* genes expression is diametrically opposite to *NRT* genes in LN and HN + IBA treatment [31]. Among the six AMT-type ammonium transporters identified in the model plant *Arabidopsis*, *AMT1.4* was expressed in the pollen [29], however, in the present study, *AMT1.4* (CSA018499) expressed in lateral roots of tea plant. *AMT1.4* was down-regulated in LRs under LN and HN + IBA treatments, and up-regulated in LN + NPA treatment. The result hints that *AMT* genes were down-regulated under low nitrogen condition and auxin treatment, and their expressions may inhibit LRs formation. In addition, the expression analysis of *NRT*, *AMT* and *NPF* family genes revealed that there exists a synergistic relationship between auxin and nitrogen signaling towards LR development. These genes contribute to N utilization efficiency exploration and provide gene reference for selection of high nitrogen-efficient varieties of tea plants. This can also contribute to the exploration of regulation of LRs formation through regulating nitrogen and auxin signal pathway.

### Plant hormone metabolism and signaling pathways

The plant hormone, auxin is critical for plant growth and development processes, and plays its regulatory role mainly by inducing expression of early auxin response genes including *Aux/IAA*, *GH3* and *SAUR*. *GH3* combines free auxin with disparate acid; therefore, overexpression of *GH3* would result in expression of severe auxin-deficient phenotypes [32, 33]. In the present study, one *GH3* gene was up-regulated under LN treatment but downregulated under LN + NPA treatment, while the HN + IBA treatment regulated *GH3* genes. In addition, Aux/IAA proteins play the role of transcriptional repressors by heterodimerizing with auxin response factor (ARF) transcription factors, while ARF family acts as key regulator of root development [34, 35]. At low auxin concentration, Aux/IAA proteins could restrain transcriptional activation of ARF proteins; thereby, preventing response genes transcription of auxin. But higher auxin concentration could induce *ARF* genes expression and then promote LRs development [35]. Under LN treatment, the Aux/IAA proteins genes (CSA031541, MSTRG.7473) were down-regulated, while auxin response factor 2 (*ARF2*) gene (CSA011327) was up-regulated. It is shown that under LN condition, the LRs’ auxin concentration would be increased, and then inhibit *Aux/IAA* genes expression and improve *ARF* genes expression to induce LRs development. Moreover, under LN + NPA treatment, the *ARF2* gene (CSA011327) was down-regulated, while *ARF2* gene (CSA012843) was up-regulated under HN + IBA treatment. This; therefore, indicates that low nitrogen and auxin treatments could improve *ARFs* expression and enhance LRs formation.

Cytokinin is important for plants proliferation, plants cell division, secondary metabolism, and regulation of plants shoot and roots development [36]. Other researchers have shown that some nitrogen signals are substituted by cytokinins as local and long-distance signal, and; thus, various genes were regulated by these plant hormones, including metabolism, growth development and nutrient absorption [37]. High cytokinin content is reported to improve shoot development while high auxin content enhances root formation [38]. Therefore, there exist an important signaling pathway among nitrogen, auxin and cytokinin in the regulation of plant development. The present study identified DEGs involved in cytokinin metabolism. Under LN treatment, one adenylate isopentenyltransferase (CAS006753) gene which induces cytokinin biosynthesis was down-regulated, while two cytokinin dehydrogenase genes which inhibit cytokinin biosynthesis were up-regulated. Under LN + NPA treatment, one cytokinin dehydrogenase gene (CAS011288) was down-regulated, but expressed in LN treatment. Under HN + IBA treatment, cytokinin hydroxylases gene (CAS017731) which induces cytokinin biosynthesis was down-regulated, while 7 cytokinin dehydrogenase genes were up-regulated. The RP-UPLC technique revealed that cytokinin concentration increased with increasing nitrogen concentration and decreased with exogenous auxin treatment. It suggests that LN treatment and auxin treatment could inhibit cytokinin biosynthesis, while high auxin condition induces LRs formation in tea plants.

Ethylene is connected to plant’s physiological and morphological responses to nitrogen deficiency, and nitrate transporters *NRT1.1* and *NRT2.1* are also sensitive to ethylene [39, 40]. Under low external nitrate concentration, *NRT2.1* induces and promotes ethylene biosynthesis and signaling activity [41]. Auxin and ethylene signaling pathways show specific regulation of plants growth and development, such as root elongation and root hair formation [42]. Studies also suggest that ethylene might stimulate localized auxin biosynthesis [43]. In the present study, ethylene biosynthesis varied with different levels of nitrogen treatment. Under LN, ethylene biosynthesis genes were up-regulated, but down-regulated under HN treatment. Under LN + NPA treatment, 3 ethylene biosynthesis genes were down-regulated while 14 genes were up-regulated under HN + IBA treatment. This; therefore, suggests that LN treatment improves ethylene biosynthesis; thereby, promoting auxin response genes expression and LRs formation, while HN treatment down-regulates ethylene biosynthesis; thus, inhibits LRs formation. Comparison between HN + IBA and LN + NPA treatments revealed that auxin could promote ethylene biosynthesis and stimulate LRs formation. These results; therefore, clearly indicates that auxin and nitrogen could regulate tea plant LRs formation through ethylene biosynthesis pathway.

### Transcription factors

Transcription factors (TFs) control the expression of stress resistance genes [44]. Many TF families such as *NAC*, *MYB*, *MADS-box* and *WRKY* have been explored [45], and these families can regulate cell division and expansion, lateral root development and secondary cell wall biosynthesis. Several TFs have been expressed in plants exposed to N-deficient situations [46, 47].

R2R3-MYB is reported to be the most abundant MYB protein. MYBs46/83 is speculated to be the prime regulator of secondary cell wall biosynthesis, while *AtMYB58* specifically activates lignin biosynthesis, as regulated by *AtMYB46* [48]. Therefore, MYBs expression would thicken the secondary cell wall and inhibit cells division and elongation [49, 50]. In the present study, the P-type R2R3 MYB protein (*MYB83*) homologous gene was down-regulated under LN and HN + IBA treatment, but up-regulated with NPA treatment. This indicates that LN treatment could inhibit *MYB* genes expression and restrain secondary cell wall biosynthesis, thus, regulating roots development. Similar inference can be made with auxin treatment. This also confirms the hypothesis that accumulation of auxin can be promoted under low nitrogen conditions in LRs of tea plant.

*NAC* transcription factors are important for plant growth as they regulate plants cell division, lateral root development and secondary cell wall biosynthesis [51, 52]. A few *NAC* genes have been identified as key and effective regulation factor in auxin signaling pathway which directly affect LRs development [53, 54]*.* In the present study, 7 *NAC* DEGs were expressed under LN treatment; 43 *NAC* DEGs were expressed under HN + IBA treatment and most of them were up-regulated. Similarly, DEGs of *NAC* also up-regulated under LN + NPA treatment. Consistent with previous reports, the present study; therefore, revealed that *NAC* genes could be induced by nitrogen treatment and auxin treatment to regulate tea plants LRs formation; however, the detail signaling pathway still needs further exploration.

MADS-box TFs control plants root, flower and fruit development [55, 56]. Previous study has shown that *AGL21* is induced by N-deprivation, and auxin also promotes *AGL21*, while AGL21 proteins interact with ANR1 (AGL44) to mediate LRs development [57, 58]. In the present study, 12 MADS-box protein genes were down-regulated under LN treatment. Under HN + IBA treatment, there are 19 DEGs. Also, *AGL* genes were up-regulated under LN + NPA treatment. Thus, it can be suggested that the expression of MADS-box protein genes in tea plant might differ from that of *Arabidopsis thaliana*.

WRKY transcription factors are involved in various plant developmental processes, such as biological and abiotic stresses, and seed germination and dormancy [59]. WRKY TF is a major player in plant’s innate immune system. Beet cyst nematode is reported to regulate WRKY TF genes expression to enhance roots development in *Arabidopsis thaliana* [60]. In a previous study, WRKY TF families were induced under N-deficient condition [61]. In the current study, WRKY TF family genes were induced by various levels of nitrogen treatments (Table S2). This reveals that WRKY TF family genes could be significantly induced by auxin signaling, and; thus, take part in nitrogen metabolism under various nitrogen conditions. It can also be deduced that TFs play important roles in nitrogen and auxin network in tea plant LRs formation. It provides a insight to explore the involvement of WRKY TF family gene via nitrogen and auxin signaling pathway in LRs formation in tea plant.

### Glutathione metabolism

KEGG analysis revealed a significant change in glutathione metabolism in all treatments. GSH-dependent developmental pathway induces and sustains cell division during root development, and regulates auxin transport and evolution [61, 62]. Glutathione also acts as thiol/disulfide buffer. It can regulate the balance between GSH (reduced form) and GSSG (oxidized form) by GSH oxidation through reactive oxygen species, and GSSG reduction through glutathione reductase [62]. Exogenous GSSG could not induce roots in normal conditions but promotes root development under auxin treatment [63]; therefore, auxin and GSSG interaction would regulate plants roots development. It is reported that the reduction ratio of GSH/GSSG inhibits lateral roots in the presence of auxin [64]. Glutathione S-transferases (GSTs) transforms GSH to GSSG, while glutathione reductase (GR) induces the reduction of GSSG into GSH [65]. In the present study, 8 GSTs genes were up-regulated and 12 GST genes were down-regulated with LN treatment. Under HN condition, 14 GSTs genes were down-regulated, while 5 GSTs genes were up-regulated. The IBA treatment up-regulated 5 glutathione reductase genes and 65 GSTs genes. Finally, 37 GSTs genes were up-regulated under NPA treatment. Nitrogen and auxin treatments annotated many DEGs in glutathione metabolism, both treatments could affect GSH/GSSG ratio to regulate LRs formation in tea plant; however, the specific adjustment mechanism is still vague and requires further research.

### F-box protein

F-box proteins are important components of proteasome pathway and participate in cellular functions such as auxin receptor (TIR1), which mediates transcriptional response to auxin in a F-box protein [66]. Many researches have shown that adventitious roots formation accompanies soluble and insoluble carbohydrates accumulation, and the *At1g23390* is shown to be related to such metabolism [67, 68]. When tea plants were treatment with IBA, a F-box/kelch gene similar to *AT1g23390* was identified and this suggests a complex regulatory network during adventitious roots development [64]. In the current study, *AT1g23390* (CAS036587) was up-regulated by HN + IBA treatment, and its homogenous gene was down-regulated by LN + NPA treatment, but up-regulated by LN treatment. It depicts that this gene could be induced by nitrogen treatment, and its function was similar with auxin treatment in root development. It also indicates that LN treatment could induce auxin production in tea plant LRs. Hence, the putative role of this gene might be to regulate LRs formation through N and auxin signaling pathway in tea plants.

## Conclusions

**Fig. 10:**
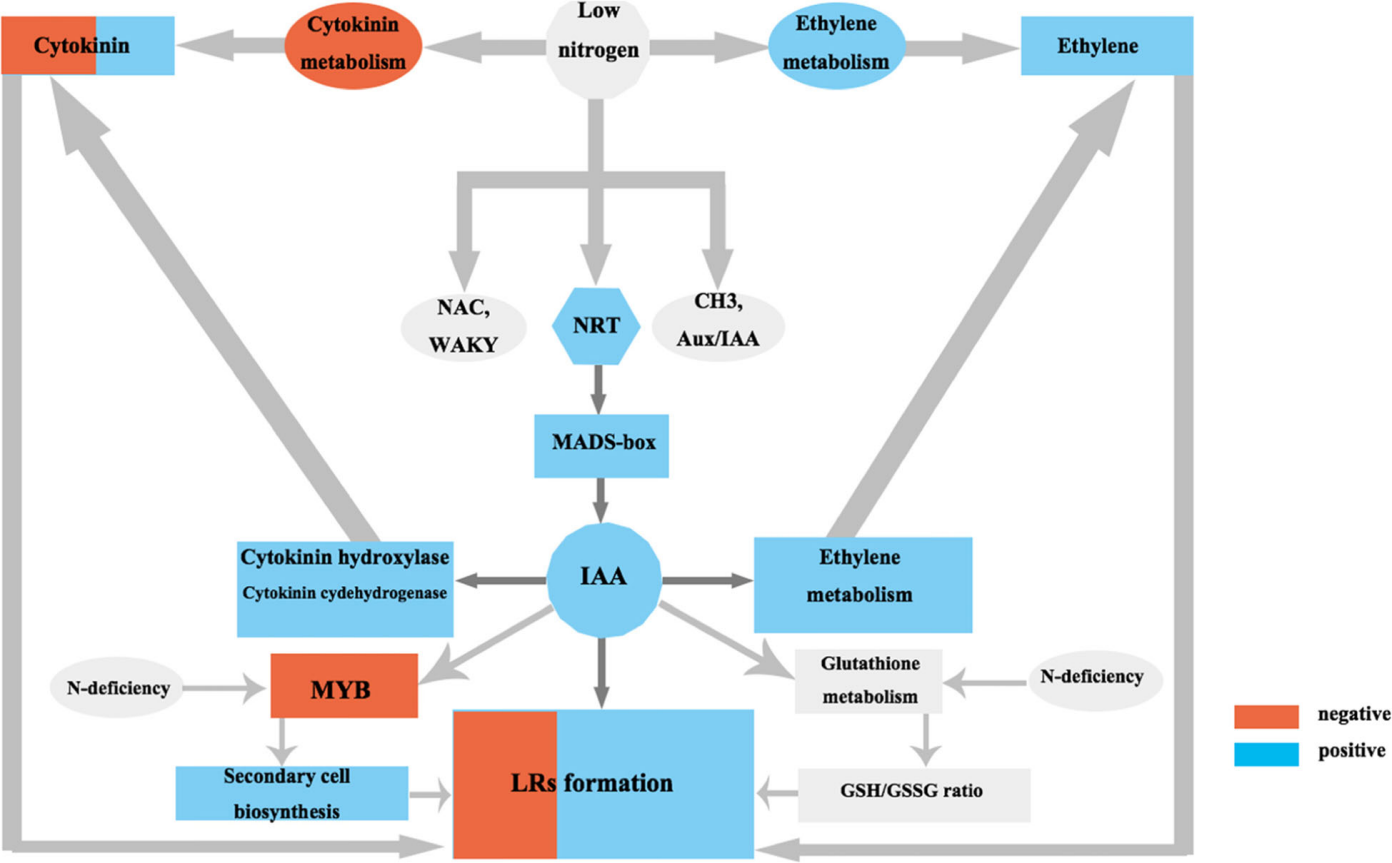
Multiple signaling pathways regulating the formation of lateral roots to N deficiency

In this study, transcriptome analysis of LRs of tea plant (*Camellia sinensis*) treated with low and high concentrations of nitrogen, indole-3-butyric acid (IBA) and *N − 1-naphthylphthalamic* acid (NPA) was carried out to reveal the function of auxin and nitrogen in LRs formation and development. Nitrogen deficiency induced the expression of *NRT* genes, thus, increasing the production and accumulation of auxin, promoting lateral roots formation. In the process of cytokinin synthesis, nitrogen deficiency could restrain cytokinin production, and the auxin accumulation and synthesis could control cytokinin production by promoting cytokinin hydroxylase and cytokinin dehydrogenase to improve auxin/cytokinin ratio, and further improve LRs formation and development. Nitrogen deficiency and auxin accumulation and synthesis also regulate growth and development of lateral roots by promoting metabolic synthesis of ethylene. Auxin and N deficiency would inhibit secondary cell wall biosynthesis by inhibiting *MYB* genes expression to facilitate lateral roots development. Auxin and N deficiency also synergistically control the GSH/GSSG ratio by effecting glutathione metabolism. TF families like *NAC*, *WRKY* and *MAD-box* genes were affected by nitrogen and auxin treatments, and similar effects were observed in early auxin response genes including *Aux/IAA* and *GH3* (Fig. 10). The results in this study are meaningful in building an overall regulation network, but require additional genetic and physiological data to realize this. This study would offer a foundation for further exploration into LRs formation, and also accelerate genomic studies on tea plant.

## Methods

### Plant materials

Purebred tea seeds (*Camellia sinensis* cv. Fuding dabai) collected from Fujian Province Lianfeng Tea Co., Ltd. were surface-sterilized and pre-grown in a liquid medium for 6 weeks. The growth medium contained 0.75 mM (NH4)_2_SO_4_, 0.25 mM Ca (NO_3_)_2•_4(H_2_O)_3_, 0.05 mM KH_2_PO_4_, 0.35 mM K_2_SO_4_, 0.395 mM CaCl_2_, 0.21 mM MgSO_4_, 35.0 μM NaFeEDTA, 46.1 μM H_3_BO_3_, 2.0 μM MnSO_4_, 0.3 μM CuSO_4_, 2.0 μM ZnSO_4_ and 0.5 μM Na_2_MoSO_4_. The basic nitrogen (N) nutrient solution was set into four levels, including a gradient N concentration of 0 mM (labelled as no nitrogen, NN), 0.25 mM (labelled as low nitrogen, LN), 1 mM (labelled as control, CK), and 2.5 mM (labelled as high nitrogen, HN). The control experiment (1 mM) was supplemented with 0.75 mM ammonium and 0.25 mM nitrate using (NH_4_)_2_SO_4_ and Ca (NO_3_)_2•_4(H_2_O) respectively, which the best combination of N concentration for seedlings growth.

The seedlings were grown in a growth chamber at 28/25 °C (day/night), 75% relative humidity, 16/8 h (light/darkness) photoperiod, and 300 μmol^− 2^ s^− 1^ light intensity. The growth medium was replaced with the same fresh liquid medium every 3–5 days. After 10 weeks of treatment when seedlings have grown LRs, the growth medium with high (2.5 mM) and low (0.25 mM) nitrogen concentrations were supplemented with 0.4 mM IBA and 20 μM NPA, respectively. Then after 24 h, the LRs treated with 0.25, 1, 2.5 mM nitrogen, 2.5 mM nitrogen + 0.4 mM IBA and 0.25 mM nitrogen + 20 μM NPA were harvested and frozen in liquid nitrogen for RNA extraction. To acquire the LRs from the tea seedlings, the seedlings were treated with 0.25 mM, 1 mM and 2.5 mM nitrogen respectively for 10 weeks, and then the tea seedlings cultured with the 2.5 mM nitrogen were further treatment with 10 μM IBA for 8 weeks to harvest lateral roots. Finally, the four kinds of LRs obtained from the collected samples were used to measure hormones using RP-UPLC examination and phenotypic observation. The tea seedlings cultured with the 0.25 mM nitrogen were further treatment with 10 μM NPA for 4 weeks to be only used for phenotypic observation.

### Lateral roots number and length analysis

The lateral roots of each tea plant were cut down and laid flat on a flat plate. The WinRHIZO root analysis system (Regent Instruments, Inc., Canada) was used to scan the lateral roots and record the total number and length of the lateral roots of each tea plant. Three biological replicates were used per each sample and each measurement was replicated three times.

### RNA isolation, library construction and RNA sequencing

Total RNA was isolated from LRs of tea seedlings using plant RNA extraction kit with DNase (TIANDZ, Inc., Beijing, China) according to the manufacturer’s protocol. The quantity and quality of the RNA samples were determined by using NanoDrop2000 Spectrophotometer (Thermo Fisher Scientific, USA), 1.2% agarose gel electrophoresis, and Agilent 2100 Bioanalyzer (Agilent Technologies, Inc., Santa Clara, CA, USA). Quality RNA samples were used for library construction and sequencing using PE150 using Illumina HiSeq platform. A total of eighteen libraries were constructed and sequenced. The experiment was replicated three times. All sequenced data have been deposited into NCBI’s Sequence Read Archive under the GenBank accession number SRA number SUB6669244.

### Sequencing data analysis

Raw reads were cleaned by removing adaptor sequence, ambiguous reads (‘N’>10%), and low-quality reads (i.e., where more than 50% of bases in a read had a quality value Q ≤ 5) using an in-house perl script. High-quality clean reads were mapped to the latest version (CAS institute of Botany, Kunming) of tea plant reference genome (http://www.plantkingdomgdb.com/tea_tree) using HISAT2 software [69, 70].

### Functional annotation and pathway analysis

All mapped genes and unmapped genes obtained in this study were annotated using public databases including NR database (http://www.ncbi.nlm.nih.gov/), Swiss-Prot database (http://www.expasy.ch/sprot), COG (http://www.ncbi.nlm.nih.gov/COG/) and Pfam databases (http://pfam.xfam.org/) using BLASTX (http://blast.ncbi.nlm.nih.gov/Blast.cgi). Gene Ontology (GO) functional classification for all annotated genes was analysed using WEGO software [71]. A Python script was used to retrieve Kyoto Encyclopedia of Genes and Genomes (KEGG) annotation from blast results. GO enrichment analysis of DEGs were performed using Singular Enrichment Analysis (SEA) method with *P* < 0.01 and FDR < 0.05 by agriGO. The hypergeometric Fisher exact test (*P* < 0.01) and Benjamini (FDR < 0.05) were performed to detect statistically significant enrichments of KEGG pathway. GO and KEGG enrichment analyses were performed using the whole tea tree transcriptome setting as reference.

### Pearson correlation analysis

According to the amount of gene expression in the samples, the correlation coefficient between samples was calculated to determine the sample correlation. For the project of biological duplication, the success of biological duplication was evaluated by the correlation of samples. Pearson’s correlation coefficient R (Pearson’s correlation coefficient) was used as the evaluation index of correlation among samples [72]. The closer “R” is to 1, the stronger the correlation between the two samples. In this study, five samples were analysed for transcriptome, and each sample was repeated three times (T1-T15).

### Differential expression analysis

Fragments per kb per million reads (FPKM) method was used to quantify the expression levels of transcripts. The DESeq2 package was used to identify DEGs [73]. The FDR ≤ 0.01 and the absolute value of log_2_ ratio ≥ 1 were set as thresholds value for significant differential gene expression between two samples.

### Reversed phase ultra performance liquid chromatography (RP-UPLC) analysis

The lateral roots were extracted with methanol for auxin concentration determination. The extracted samples were analysed using RP-UPLC with the follows conditions: Hypersil ODS C18 column (250 mm X 4.0 mm, 5 μm); methanol and ultrapure water (0.5% glacial acetic acid) as mobile phase and gradient elution; column temperature of 35 °C, injection volume of 15 μL, flow rate of 1 mL/min, and detection wavelength of 254 nm. External standard calibration curve method was employed for the quantitative analysis. For standard preparation and standard curve construction, standard IAA and ZT were dissolved in 50% chromatographic methanol. Peak area (Y) was used as linear regression curve for the mass (X, nmol) to obtain the linear range of the regression equation, standard curve, and detection limits. The concentration gradient was 12.5, 25, 50, 100 and 200 ng/mL. Each measurement was replicated three times.

### Quantitative RT-PCR (qRT-PCR) analysis

Total RNA was extracted using plant RNA extraction kit with DNase (TIANDZ Inc., Beijing, China) according to the manufacturer’s protocol. First-strand cDNA was synthesized from 2 μg of total RNA using Prime Script RT Reagent Kit (Takara, Japan). The qRT-PCR reaction was performed in a 96-well plates using Bio-Rad Real-time thermal cycler CFX96 with SYBR Premix ExTaq™ Kit (Takara, Dalian, China). The glyceraldehyde-3-phosphate dehydrogenase gene (*GAPDH*) and *ACTIN* of tea plant were used as reference genes. The relative mRNA expression levels were calculated using the 2^-ΔΔCt^ method [74]. Three biological replicates were performed for each sample. Microsoft Excel 2016 and Sigmaplot 13.0 (Systat Software, Canda) were used for statistical analysis. The difference between the two samples was assessed using the student t-test method, *P* < 0.05 was considered as significantly different in content. The qPCR primers of differentially expressed genes were designed using NCBI primer-BLAST (https://www.ncbi.nlm.nih.gov/tools/primer-blast/). Information on genes and primer sequences used in this study are listed in Table S1.

## Supplementary information

**Additional file 1 Figure S1**. GO Analysis of DEGs between control (CK) and low nitrogen (LN) treatments. **Figure S2**. GO Analysis of DEGs between high nitrogen (HN) and control (CK) treatments. **Figure S3**. GO Analysis of DEGs between low nitrogen (LN) and LN + NPA treatments. **Figure S4**. GO Analysis of DEGs between high nitrogen (HN) and HN + IBA treatments. **Figure S5**. KEGG Analysis of DEGs between control (CK) and low nitrogen (LN) treatments. **Figure S6**. KEGG Analysis of DEGs between high nitrogen (HN) and control (CK) treatments. **Figure S7**. KEGG Analysis of DEGs between low nitrogen (LN) and LN + NPA treatments. **Figure S8**. KEGG Analysis of DEGs between high nitrogen (HN) and HN + IBA treatments.

**Additional file 2 Table S1.** Annotation and primers for genes verified by qRT-PCR. **Table S2a.** DEGs related to nitrogen metabolism, plant hormone signal transduction, glutathione metabolism and transcription factors (TFs) between control (CK) and low nitrogen (LN). **Table S2b.** DEGs related to nitrogen metabolism, plant hormone signal transduction, glutathione metabolism and transcription factors (TFs) between control (CK) and high nitrogen (HN). **Table S2c.** DEGs related to nitrogen metabolism, plant hormone signal transduction, glutathione metabolism and transcription factors (TFs) between LN and LN + NPA. **Table S2d.** DEGs related to nitrogen metabolism, plant hormone signal transduction, glutathione metabolism and transcription factors (TFs) between HN and HN + IBA.

## Data Availability

All data sustaining the results in this study are included in this article or its information files. Other datasets generated during this study are available upon reasonable request from the corresponding author (Xinghui Li).

## Abbreviations

LRs: Lateral roots
IBA: indole-3-butyric acid
NPA: *N-1-naphthylphthalamic* acid
RP-UPLC: Eversed phase ultra performance liquid chromatography
NN: No nitrogen
LN: Low nitrogen
HN: High nitrogen
NRTs: Nitrate transporter genes
AFB3: AUXIN SIGNALING F-BOX 3
TAR2: Tryptophan aminotransferase related 2
ABA: Abscisic acid
AMT: Ammonium transporter
ARF2: Auxin response factor 2
GSTs: Glutathione S-transferases
Trp: Tryptophan
TAA1: Tryptophan aminotransferase of Arabidopsis1
TAR4: Tryptophan aminotransferase related 4
TFs: Transcription factors
NAC: NAM, ATAF1/2 and CUC2
