# Supplementary material for: A novel insight into nitrogen and auxin signaling in lateral root formation in tea plant [Camellia sinensis (L.) O. Kuntze]
Source: BMC Plant Biol. 2020 May 24;20:232. doi: 10.1186/s12870-020-02448-7 (PMC7247184; doi:10.1186/s12870-020-02448-7)
Supplement: Supplementary file 1 — Additional file 1 Figure S1. GO Analysis of DEGs between control (CK) and low nitrogen (LN) treatments. Figure S2. GO Analysis of DEGs between high nitrogen (HN) and control (CK) treatments. Figure S3. GO Analysis of DEGs between low nitrogen (LN) and LN + NPA treatments. Figure S4. GO Analysis of DEGs between high nitrogen (HN) and HN + IBA treatments. Figure S5. KEGG Analysis of DEGs between control (CK) and low nitrogen (LN) treatments. Figure S6. KEGG Analysis of DEGs between high nitrogen (HN) and control (CK) treatments. Figure S7. KEGG Analysis of DEGs between low nitrogen (LN) and LN + NPA treatments. Figure S8. KEGG Analysis of DEGs between high nitrogen (HN) and HN + IBA treatments. [file 12870_2020_2448_MOESM1_ESM.docx]

Figure S1. GO Analysis of DEGs between control (CK) and low nitrogen (LN) treatments

Figure S2. GO Analysis of DEGs between high nitrogen (HN) and control (CK) treatments

Figure S3. GO Analysis of DEGs between low nitrogen (LN) and LN + NPA treatments

Figure S4. GO Analysis of DEGs between high nitrogen (HN) and HN + IBA treatments

Figure S5. KEGG Analysis of DEGs between control (CK) and low nitrogen (LN) treatments

Figure S6. KEGG Analysis of DEGs between high nitrogen (HN) and control (CK) treatments

Figure S7. KEGG Analysis of DEGs between low nitrogen (LN) and LN + NPA treatments

Figure S8. KEGG Analysis of DEGs between high nitrogen (HN) and HN + IBA treatments
