# Supplementary material for: A novel insight into nitrogen and auxin signaling in lateral root formation in tea plant [Camellia sinensis (L.) O. Kuntze]
Source: BMC Plant Biol. 2020 May 24;20:232. doi: 10.1186/s12870-020-02448-7 (PMC7247184; doi:10.1186/s12870-020-02448-7)
Supplement: Supplementary file 2 — Additional file 2 Table S1. Annotation and primers for genes verified by qRT-PCR. Table S2a. DEGs related to nitrogen metabolism, plant hormone signal transduction, glutathione metabolism and transcription factors (TFs) between control (CK) and low nitrogen (LN). Table S2b. DEGs related to nitrogen metabolism, plant hormone signal transduction, glutathione metabolism and transcription factors (TFs) between control (CK) and high nitrogen (HN). Table S2c. DEGs related to nitrogen metabolism, plant hormone signal transduction, glutathione metabolism and transcription factors (TFs) between LN and LN + NPA. Table S2d. DEGs related to nitrogen metabolism, plant hormone signal transduction, glutathione metabolism and transcription factors (TFs) between HN and HN + IBA. [file 12870_2020_2448_MOESM2_ESM.docx]

Table S1 | Annotation and primers for genes verified by qRT-PCR.

| Gene ID | Log2 (Fold change) | | | | | | | | Forward primer (5’-3’) | | | | Reverse primer (5’-3’) | | | Description | | |
| --- | --- | --- | --- | --- | --- | --- | --- | --- | --- | --- | --- | --- | --- | --- | --- | --- | --- | --- |
|  | CK vs LN | | CK vs HN | | LN vs LN+NPA | | HN vs HN+IBA | | |  |  | | |  | | |  |  |
| CSA011051 | 1.84 | 1.53 | | -3.08 | | -3.24 | | AACTCAGATCATCATGCCTTAC | | | | TCTACTCCGAAACAATACCC | | | High affinity nitrate transporter 2.5 | | |  |
| MSTRG.51865 | 1.51 | 1.23 | | -2.73 | | -2.72 | | CAGTTTCTGGTGCTGTTT | | | | GTTGTATTGCTCCTCCTC | | | High affinity nitrate transporter 2.4 | | |  |
| CSA015778 | - | - | | - | | -2.56 | | GCAGATGGGTGACAGGAC | | | | GGGTAAGACGAGTAATACGG | | | Tryptophan aminotransferase-related protein 2 | | |  |
| CSA018499 | -2.32 | - | | 2.61 | | -1.71 | | GCACGACCTAATACCCTCC | | | | CAGCCAAACCATAACAAGAA | | | Ammonium transporter 1 member 4 | | |  |
| CSA011327 | 3.86 | - | | -4.11 | | - | | GCAGCAGTTCTTTGAGTGG | | | | CATCGCCTGTTCAATATGTC | | | Auxin response factor 2 | | |  |
| CSA006753 | -1.33 | - | | - | | -3.83 | | CATTCTCAGCAGCCATCA | | | | ACCACCACCTTGTCCTTG | | | Adenylate isopentenyltransferase | | |  |
| CSA011288 | 1.16 | - | | -2.00 | | - | | AGTGGAATGGAAATGCAGGTT | | | | CCGCCAACTATGAGATGTAGGTAA | | | Cytokinin dehydrogenase | | |  |
| CSA017731 | -2.62 | - | | - | | - | | CTGAGTGGCAAGATAAGGT | | | | GTGAATGGCTAGAACTGGA | | | Cytokinin hydroxylase | | |  |
| MSTRG.31932 | 1.71 | - | | -1.31 | | - | | CCGTCCCGATCCGTGTATT | | | | AACCGCCGTCAACGAACT | | | F-box/kelch protein | | |  |
| CSA036587 | - | - | | - | | 1.17 | | TATGGTGGTGGCTCAAGAAG | | | | GAGGGTGGAGATTTGCTGT | | | F-box/kelch protein | | |  |
| CSA012447 | -1.46 | -1.26 | | 2.74 | | 9.59 | | AAGCTCATAGGTGGTTATTC | | | | GGCTTGTTGTGGTGGATA | | | Glutathione S-transferase | | |  |

“ – ” represents no significant difference in gene expression. LN (0.25 mM nitrogen for 10 weeks + 24 h), CK (the control, 1 mM nitrogen for 10 weeks + 24 h), HN (2.5mM nitrogen for 10 weeks + 24 h), LN + NPA (0.25 mM nitrogen for 10 weeks, and then cultured with 0.25 mM nitrogen + 10 μM NPA for 24 h), HN + IBA (2.5 mM nitrogen for 10 weeks, and then cultured with 2.5 mM nitrogen + 10 μM IBA for 24 h).

Table S2a. DEGs related to nitrogen metabolism, plant hormone signal transduction, Glutathione metabolism and transcription factors (TFs) between control (CK) and low nitrogen (LN)

| Gene ID | log2 Ratio (LN/CK) | Up-Down Regulation (LN/CK) | Gene annotation | |
| --- | --- | --- | --- | --- |
| \| CSA018239 \| \| --- \| \| CSA023726 \| \| CSA033843 \| \| CSA019587 \| \| CSA003776 \| \| CSA027055 \| \| CSA027057 \| \| CSA029198 \| \| CSA001095 \| \| CSA001287 \| \| CSA027056 \| \| CSA008039 \| \| CSA008038 \| \| CSA008040 \| \| CSA020541 \| \| CSA019392 \| \| CSA000189 \| \| MSTRG.51532 \| \| CSA032870 \| | \| 3.319947039 \| \| --- \| \| 2.824755576 \| \| 2.237444726 \| \| 1.910322443 \| \| 1.858224155 \| \| 1.680411298 \| \| 1.326470339 \| \| 1.267162366 \| \| 1.215227307 \| \| 1.130183035 \| \| 1.060533254 \| \| 1.052268934 \| \| 1.052268934 \| \| 1.052268934 \| \| 1.040059897 \| \| -1.253967792 \| \| -1.318213725 \| \| -1.735107113 \| \| -2.12126444 \| | up  up  up  up  up  up  up  up  up  up  up  up  up  up  up  down  down  down  down | \| Protein NRT1/ PTR FAMILY 4.5 \| \| --- \| \| Protein NRT1/ PTR FAMILY 5.5 \| \| Protein NRT1/ PTR FAMILY 5.6 \| \| Protein NRT1/ PTR FAMILY 4.5 \| \| Protein NRT1/ PTR FAMILY 4.5 \| \| Protein NRT1/ PTR FAMILY \| \| Protein NRT1/ PTR FAMILY 8.3 \| \| Protein NRT1/ PTR FAMILY 7.3 \| \| Protein NRT1/ PTR FAMILY 5.2 \| \| Protein NRT1/ PTR FAMILY 8.3 \| \| Protein NRT1/ PTR FAMILY 8.3 \| \| Protein NRT1/ PTR FAMILY 5.2 \| \| Protein NRT1/ PTR FAMILY 5.2 \| \| Protein NRT1/ PTR FAMILY 5.3 \| \| Protein NRT1/ PTR FAMILY 8.3 \| \| Protein NRT1/ PTR FAMILY 5.5 \| \| Protein NRT1/ PTR FAMILY 3.1 \| \| Protein NRT1/ PTR FAMILY 8.3 \| \| Protein NRT1/ PTR FAMILY 3.1 \| | |
| \| CSA031541 \| \| --- \| \| MSTRG.7473 \| \| CSA004419 \| \| CSA023148 \| \| CSA002098 \| | \| -1.023245913 \| \| --- \| \| -3.419902872 \| \| 1.141928895 \| \| \| 1.026136779 \| \| \| 1.020860344 \| \| | down  down  up  up  up | AUX/IAA family  AUX/IAA family  GH3 auxin-responsive promoter  GH3 auxin-responsive promoter  GH3 auxin-responsive promoter |  |
| \| CSA028278 \| \| --- \| | \| 1.161636093 \| \| --- \| | up | ethylene biosynthetic process |  |
| \| CSA002871 \| \| --- \| \| MSTRG.16244 \| \| CSA022159 \| \| MSTRG.3898 \| \| MSTRG.51720 \| \| CSA009028 \| \| CSA031833 \| \| CSA000131 \| \| MSTRG.10841 \| \| MSTRG.45862 \| \| CSA016398  CSA033075 \| | \| -1.126727587 \| \| --- \| \| -1.133580216 \| \| -1.204520628 \| \| -1.234835264 \| \| -1.305303729 \| \| -1.922739252 \| \| -2.67916958 \| \| -2.85286619 \| \| -2.868095687 \| \| -3.800984841 \| \| -5.978774432 \| \| 1.45816481 \| | down  down  down  down  down  down  down  down  down  down  down  up | MADS-box protein  MADS-box protein  MADS-box protein  MADS-box protein  MADS-box protein  MADS-box protein  MADS-box protein  MADS-box protein  MADS-box protein  MADS-box protein  MADS-box protein  NAC domain-containing protein |  |
| CSA02991 | 1.179667575 | up | NAC domain-containing protein |  |
| MSTRG.45520  CSA005671   \| CSA026809 \| \| --- \| \| MSTRG.35982 \| \| MSTRG.3755   \| CSA000819 \| \| --- \| \| MSTRG.24298 \| \| MSTRG.42422 \| \| CSA013924 \| \| CSA034770 \| \| CSA007109 \| \| CSA036012 \| \| | 1.388902748  1.084982557   \| -1.12518286 \| \| --- \| \| -1.251502589 \| \| -1.562292407 \| \| 1.816450767 \| \| \| 1.270093881 \| \| \| 1.192157953 \| \| \| 1.113086036 \| \| \| 1.064987215 \| \| \| -1.112338111 \| \| \| -1.258003276 \| \| | up  up  down  down  down  up  up  up  up  up  down  down | NAC domain-containing protein  NAC domain-containing protein  NAC domain-containing protein  NAC domain-containing protein  NAC domain-containing protein  WRKY transcription factor  WRKY transcription factor  WRKY transcription factor  WRKY transcription factor  WRKY transcription factor  WRKY transcription factor  WRKY transcription factor |  |

| \| MSTRG.14261 \| \| --- \| \| CSA024366 \| \| MSTRG.21065 \| \| MSTRG.20068 \| \| MSTRG.7154 \| \| MSTRG.51854 \| \| MSTRG.93 \| \| MSTRG.14596 \| \| CSA022978 \| \| MSTRG.26531 \| \| CSA023557 \| \| CSA011044 \| \| MSTRG.33443 \| \| CSA023543 \| \| CSA012447 \| \| CSA012448 \| \| CSA022649 \| \| CSA009742 \| \| MSTRG.6279 \| \| MSTRG.6278 \| | \| 1.783987704 \| \| --- \| \| 1.614606796 \| \| 1.39335052 \| \| 1.203023056 \| \| 1.183335966 \| \| 1.16738043 \| \| 1.102788286 \| \| 1.059506081   \| -1.008132371 \| \| --- \| \| -1.028932011 \| \| -1.078365409 \| \| -1.139890597 \| \| -1.263607894 \| \| -1.339910713 \| \| -1.467362354 \| \| -1.467362354 \| \| -1.777240075 \| \| -2.176258218 \| \| -2.633654289 \| \| -3.588988012 \| \| | up  up  up  up  up  up  up  up  down  down  down  down  down  down  down  down  down  down  down  down | Glutathione S-transferase  Glutathione S-transferase  Glutathione S-transferase  Glutathione S-transferase  Glutathione S-transferase  Glutathione S-transferase  Glutathione S-transferase  Glutathione S-transferase  Glutathione S-transferase  Glutathione S-transferase  Glutathione S-transferase  Glutathione S-transferase  Glutathione S-transferase  Glutathione S-transferase  Glutathione S-transferase  Glutathione S-transferase  Glutathione S-transferase  Glutathione S-transferase  Glutathione S-transferase  Glutathione S-transferase |
| --- | --- | --- | --- | --- | --- | --- | --- | --- | --- | --- | --- | --- | --- | --- | --- | --- | --- | --- | --- | --- | --- | --- | --- | --- | --- | --- | --- | --- | --- | --- | --- | --- | --- | --- | --- | --- | --- | --- | --- | --- | --- | --- | --- |

Table S2b. DEGs related to nitrogen metabolism, plant hormone signal transduction, Glutathione metabolism and transcription factors (TFs) between control (CK) and high nitrogen (HN)

| Gene ID | log2 Ratio (HN/CK) | Up-Down Regulation (HN/CK) | Gene annotation |
| --- | --- | --- | --- |
| \| CSA013245 \| \| --- \| \| CSA012791 \| \| CSA018674 \| \| CSA018284 \| \| CSA001287 \| \| CSA001095 \| \| CSA005114 \| \| CSA000511 \| \| CSA000512 \| \| CSA000510 \| \| CSA027055 \| \| CSA018435 \| \| MSTRG.49377 \| \| CSA029198 \| \| CSA023726 \| \| CSA022524 \| \| MSTRG.7473 \| \| MSTRG.45542   \| CSA009976 \| \| --- \| \| CSA033915 \| \| CSA013292  MSTRG.10434  CSA016398  CSA022556   \| CSA026809 \| \| --- \| \| CSA003142 \| \| CSA019155 \| \| \| \| MSTRG.526 \| \| --- \| \| MSTRG.31415 \| \| MSTRG.28367 \| \| CSA013924 \| \| CSA008877 \| \| CSA020053 \| \| CSA017015 \| \| CSA027168 \| \| \| \| CSA010224 \| \| --- \| \| CSA017239 \| \| MSTRG.33443 \| \| MSTRG.34202 \| \| MSTRG.24788 \| \| CSA033203 \| \| CSA012447 \| \| CSA012448 \| \| CSA023543 \| \| MSTRG.6336 \| \| MSTRG.47061 \| \| MSTRG.17369 \| \| CSA006423 \| \| CSA017213 \| \| MSTRG.14261 \| \| CSA022649 \| \| MSTRG.14596 \| \| CSA016859 \| \| MSTRG.51854 \| \| \| | \| -1.399632037 \| \| --- \| \| -1.243495706 \| \| 1.015174096 \| \| 1.02103549 \| \| 1.106309929 \| \| 1.144720557 \| \| 1.169598433 \| \| 1.170012128 \| \| 1.170012128 \| \| 1.170012128 \| \| 1.174255056 \| \| 1.186144296 \| \| 1.420613991 \| \| 1.427761388 \| \| 2.954496987 \| \| -3.750805519 \| \| -2.533077057 \| \| -1.014715458   \| 1.018007268 \| \| --- \| \| 1.059158926 \| \| 1.268473639 \| \| -1.640698857  -2.445666968  1.116028756   \| -1.096437731 \| \| --- \| \| 1.095742794 \| \| 1.144597569   \| -1.086942326 \| \| --- \| \| -1.030297591 \| \| -1.021287085 \| \| 1.054893745 \| \| 1.092383115 \| \| 1.203198119 \| \| 1.433242981 \| \| 1.711708727   \| -2.592915262 \| \| --- \| \| -2.554059172 \| \| -2.034298373 \| \| -1.511764849 \| \| -1.329735962 \| \| -1.283285487 \| \| -1.267574807 \| \| -1.267574807 \| \| -1.192310681 \| \| -1.159071714 \| \| -1.074800371 \| \| -1.065450513 \| \| -1.048305289 \| \| -1.020313974 \| \| 1.026533974 \| \| 1.113261403 \| \| 1.140827876 \| \| 2.10220816 \| \| 2.485278832 \| \| \| \| \| | \| down \| \| --- \| \| down \| \| up \| \| up \| \| up \| \| up \| \| up \| \| up \| \| up \| \| up \| \| up \| \| up \| \| up \| \| up \| \| up \| \| down \| \| down \| \| down   \| up \| \| --- \| \| up \| \| up  down  down  up   \| down \| \| --- \| \| up \| \| up   \| down \| \| --- \| \| down \| \| down \| \| up \| \| up \| \| up \| \| up \| \| up   \| down \| \| --- \| \| down \| \| down \| \| down \| \| down \| \| down \| \| down \| \| down \| \| down \| \| down \| \| down \| \| down \| \| down \| \| down \| \| up \| \| up \| \| up \| \| up \| \| up \| \| \| \| \| | \| Protein NRT1/ PTR FAMILY 4.6 \| \| --- \| \| Protein NRT1/ PTR FAMILY 5.6 \| \| Protein NRT1/ PTR FAMILY 4.6 \| \| Protein NRT1/ PTR FAMILY 2.6 \| \| Protein NRT1/ PTR FAMILY 8.3 \| \| Protein NRT1/ PTR FAMILY 5.2 \| \| Protein NRT1/ PTR FAMILY 8.1 \| \| Protein NRT1/ PTR FAMILY 5.5 \| \| Protein NRT1/ PTR FAMILY 5.5 \| \| Protein NRT1/ PTR FAMILY 5.5 \| \| Protein NRT1/ PTR FAMILY 8.3 \| \| Protein NRT1/ PTR FAMILY 3.1 \| \| Protein NRT1/ PTR FAMILY 5.5 \| \| Protein NRT1/ PTR FAMILY 7.3 \| \| Protein NRT1/ PTR FAMILY 5.5 \| \| AUX/IAA family \| \| AUX/IAA family  AUX/IAA family   \| GH3 auxin-responsive promoter \| \| --- \| \| GH3 auxin-responsive promoter  GH3 auxin-responsive promote  ethylene biosynthetic process  MADS-box protein  MADS-box protein   \| NAC domain-containing protein \| \| --- \| \| NAC domain-containing protein \| \| NAC domain-containing protein  WRKY transcription factor  WRKY transcription factor  WRKY transcription factor  WRKY transcription factor  WRKY transcription factor  WRKY transcription factor  WRKY transcription factor  WRKY transcription factor  Glutathione S-transferase  Glutathione S-transferase  Glutathione S-transferase  Glutathione S-transferase  Glutathione S-transferase  Glutathione S-transferase  Glutathione S-transferase  Glutathione S-transferase  Glutathione S-transferase  Glutathione S-transferase  Glutathione S-transferase  Glutathione S-transferase  Glutathione S-transferase  Glutathione S-transferase  Glutathione S-transferase  Glutathione S-transferase  Glutathione S-transferase  Glutathione S-transferase  Glutathione S-transferase \| \| \| |

Table S2c. DEGs related to nitrogen metabolism, plant hormone signal transduction, Glutathione metabolism and transcription factors (TFs) between LN and LN + NPA

| Gene ID | log2 Ratio (LN/LN+NPA) | Up-Down Regulation (LN/LN+NPA) | Gene annotation |
| --- | --- | --- | --- |
| \| CSA018239 \| \| --- \| \| CSA023726 \| \| CSA019587 \| \| CSA013711 \| \| CSA018284 \| \| CSA029605 \| \| CSA023727 \| \| CSA023728 \| \| CSA003776 \| \| MSTRG.49377 \| \| MSTRG.20513 \| \| CSA020711 \| \| MSTRG.51532 \| \| CSA016141 \| \| CSA018435 \| \| CSA025674 \| \| CSA004375 \| \| CSA000188 \| \| CSA000189 \| \| CSA004480 \| | \| -3.163575991 \| \| --- \| \| -2.096298087 \| \| -1.792450922 \| \| -1.608639237 \| \| -1.374374048 \| \| -1.239908062 \| \| -1.160254916 \| \| -1.160254916 \| \| -1.036197753 \| \| 1.029836601 \| \| 1.038965983 \| \| 1.144834294 \| \| 1.355943687 \| \| 1.407477502 \| \| 1.40883527 \| \| 1.665461939 \| \| 1.841889708 \| \| 1.864634879 \| \| 2.588794847 \| \| 2.600589937 \| | \| down \| \| --- \| \| down \| \| down \| \| down \| \| down \| \| down \| \| down \| \| down \| \| down \| \| up \| \| up \| \| up \| \| up \| \| up \| \| up \| \| up \| \| up \| \| up \| \| up \| \| up \| | \| Protein NRT1/ PTR FAMILY 4.5 \| \| --- \| \| Protein NRT1/ PTR FAMILY 5.5 \| \| Protein NRT1/ PTR FAMILY 4 \| \| Protein NRT1/ PTR FAMILY 7.3 \| \| Protein NRT1/ PTR FAMILY 2.6 \| \| Protein NRT1/ PTR FAMILY 7.3 \| \| Protein NRT1/ PTR FAMILY 5.5 \| \| Protein NRT1/ PTR FAMILY 5.5 \| \| Protein NRT1/ PTR FAMILY 4.5 \| \| Protein NRT1/ PTR FAMILY 5.5 \| \| Protein NRT1/ PTR FAMILY 1.2 \| \| Protein NRT1/ PTR FAMILY 4.5 \| \| Protein NRT1/ PTR FAMILY 8.3 \| \| Protein NRT1/ PTR FAMILY 3.1 \| \| Protein NRT1/ PTR FAMILY 3.1 \| \| Protein NRT1/ PTR FAMILY 2.13 \| \| Protein NRT1/ PTR FAMILY 3.1 \| \| Protein NRT1/ PTR FAMILY 3.1 \| \| Protein NRT1/ PTR FAMILY 3.1 \| \| Protein NRT1/ PTR FAMILY 8.1 \| |
| \| CSA022524 \| \| --- \| \| CSA010141 \| \| MSTRG.17263 \| \| CSA012931 \| \| MSTRG.45542 \| \| CSA001824 \| \| CSA023080 \| | \| -3.505000153 \| \| --- \| \| -1.336869636 \| \| -1.244363905 \| \| 1.062255938 \| \| 1.214948886 \| \| 2.297489055 \| \| 2.337267551 \| | \| down \| \| --- \| \| down \| \| down \| \| up \| \| up \| \| up \| \| up \| | \| AUX/IAA family \| \| --- \| \| AUX/IAA family \| \| AUX/IAA family \| \| AUX/IAA family \| \| AUX/IAA family \| \| AUX/IAA family \| \| AUX/IAA family \| |
| \| CSA023148 \| \| --- \| \| CSA013122 \| \| CSA013292 \| | \| -1.778026282 \| \| --- \| \| 1.420929175 \| \| 2.555111234 \| | \| down \| \| --- \| \| up \| \| up \| | \| GH3 auxin-responsive promoter \| \| --- \| \| GH3 auxin-responsive promoter \| \| GH3 auxin-responsive promoter \| |
| \| CSA000345 \| \| --- \| \| CSA017586 \| \| CSA025889 \| | \| -1.682764631 \| \| --- \| \| -1.631700728 \| \| -1.320488009 \| | \| down \| \| --- \| \| down \| \| down \| | \| ethylene biosynthetic process \| \| --- \| \| ethylene biosynthetic process \| \| ethylene biosynthetic process \| |
| \| MSTRG.5546 \| \| --- \| \| MSTRG.22069 \| \| CSA009028 \| \| MSTRG.51720 \| \| MSTRG.45862 \| \| CSA016398 \| | \| -1.383894525 \| \| --- \| \| -1.362452977 \| \| 2.401281086 \| \| 2.586032199 \| \| 3.693194939 \| \| 4.891766286 \| | \| down \| \| --- \| \| down \| \| up \| \| up \| \| up \| \| up \| | \| MADS-box protein \| \| --- \| \| MADS-box protein \| \| MADS-box protein \| \| MADS-box protein \| \| MADS-box protein \| \| MADS-box protein \| |
| \| CSA032245 \| \| --- \| \| CSA001761 \| \| CSA023607 \| \| CSA003142 \| \| CSA026809 \| \| CSA015968 \| \| CSA023606 \| \| CSA031346 \| \| MSTRG.35982 \| \| CSA009919 \| \| CSA004457 \| \| CSA025566 \| | \| -1.108485426 \| \| --- \| \| -1.107782469 \| \| 1.31849931 \| \| 1.418728523 \| \| 1.518388164 \| \| 1.712899297 \| \| 2.094117255 \| \| 2.09789152 \| \| 2.256743572 \| \| 2.682077758 \| \| 2.992306321 \| \| 3.165813733 \| | \| down \| \| --- \| \| down \| \| up \| \| up \| \| up \| \| up \| \| up \| \| up \| \| up \| \| up \| \| up \| \| up \| | \| NAC domain-containing protein \| \| --- \| \| NAC domain-containing protein \| \| NAC domain-containing protein \| \| NAC domain-containing protein \| \| NAC domain-containing protein \| \| NAC domain-containing protein \| \| NAC domain-containing protein \| \| NAC domain-containing protein \| \| NAC domain-containing protein \| \| NAC domain-containing protein \| \| NAC domain-containing protein  NAC domain-containing protein \| |
| \| CSA034770 \| \| --- \| \| MSTRG.43835 \| \| CSA014257 \| \| CSA013924 \| \| MSTRG.24298 \| \| CSA021419 \| \| MSTRG.47422 \| \| MSTRG.47297 \| \| MSTRG.26226 \| \| CSA029390 \| \| MSTRG.51054 \| \| MSTRG.12649 \| \| CSA027168 \| \| CSA020766 \| | \| -1.426920869 \| \| --- \| \| -1.335150106 \| \| -1.296595587 \| \| -1.252458614 \| \| -1.155858014 \| \| 1.099951162 \| \| 1.118503174 \| \| 1.180215112 \| \| 1.416880728 \| \| 1.466006732 \| \| 1.482471622 \| \| 1.490362098 \| \| 1.553972618 \| \| 2.66543494 \| | \| down \| \| --- \| \| down \| \| down \| \| down \| \| down \| \| up \| \| up \| \| up \| \| up \| \| up \| \| up \| \| up \| \| up \| \| up \| | \| WRKY transcription factor \| \| --- \| \| WRKY transcription factor \| \| WRKY transcription factor \| \| WRKY transcription factor \| \| WRKY transcription factor \| \| WRKY transcription factor \| \| WRKY transcription factor \| \| WRKY transcription factor \| \| WRKY transcription factor \| \| WRKY transcription factor \| \| WRKY transcription factor \| \| WRKY transcription factor \| \| WRKY transcription factor \| \| WRKY transcription factor \| |
| \| MSTRG.21065 \| \| --- \| \| CSA024366 \| \| CSA030614 \| \| CSA032297 \| \| CSA024847 \| \| CSA024848 \| \| CSA006712 \| \| MSTRG.51854 \| \| CSA023556 \| \| MSTRG.22353 \| \| MSTRG.20068 \| \| MSTRG.35323 \| \| CSA007992 \| \| CSA011044 \| \| MSTRG.28039 \| \| CSA021873 \| \| CSA006422 \| \| CSA006421 \| \| MSTRG.14596 \| \| MSTRG.5459 \| \| MSTRG.15864 \| \| CSA022980 \| \| CSA023557 \| \| MSTRG.6279 \| \| MSTRG.50602 \| \| MSTRG.26531 \| \| CSA007383 \| \| CSA000121 \| \| CSA013391 \| \| MSTRG.37873 \| \| CSA002726 \| \| CSA023543 \| \| CSA007991 \| \| CSA007990 \| \| CSA000860 \| \| CSA020696 \| \| MSTRG.20786 \| \| MSTRG.6278 \| \| CSA011661 \| \| MSTRG.33799 \| \| CSA022649 \| \| CSA012447 \| \| CSA012448 \| \| CSA027216 \| \| MSTRG.28041 \| | \| -4.034521946 \| \| --- \| \| -3.248035034 \| \| -2.67674145 \| \| -1.609471876 \| \| -1.182252565 \| \| -1.182252565 \| \| -1.044502826 \| \| 1.172685278 \| \| 1.225641909 \| \| 1.285020116 \| \| 1.307387963 \| \| 1.330623796 \| \| 1.39331304 \| \| 1.416098811 \| \| 1.470354479 \| \| 1.485340338 \| \| 1.517847569 \| \| 1.517847569 \| \| 1.521684819 \| \| 1.539255592 \| \| 1.554390742 \| \| 1.582004839 \| \| 1.601558754 \| \| 1.680374049 \| \| 1.698967358 \| \| 1.743664825 \| \| 1.804449018 \| \| 1.854827908 \| \| 1.927637863 \| \| 1.927637863 \| \| 2.015224252 \| \| 2.028808791 \| \| 2.059808639 \| \| 2.059808639 \| \| 2.060831643 \| \| 2.231624799 \| \| 2.236704585 \| \| 2.256854519 \| \| 2.317106686 \| \| 2.478038514 \| \| 2.674846973 \| \| 2.741833392 \| \| 2.741833392 \| \| 2.753131145 \| \| 2.777410705 \| | \| down \| \| --- \| \| down \| \| down \| \| down \| \| down \| \| down \| \| down \| \| up \| \| up \| \| up \| \| up \| \| up \| \| up \| \| up \| \| up \| \| up \| \| up \| \| up \| \| up \| \| up \| \| up \| \| up \| \| up \| \| up \| \| up \| \| up \| \| up \| \| up \| \| up \| \| up \| \| up \| \| up \| \| up \| \| up \| \| up \| \| up \| \| up \| \| up \| \| up \| \| up \| \| up \| \| up \| \| up \| \| up \| \| up \| | Glutathione S-transferase  Glutathione S-transferase  Glutathione S-transferase  Glutathione S-transferase  Glutathione S-transferase  Glutathione S-transferase  Glutathione S-transferase  Glutathione S-transferase  Glutathione S-transferase  Glutathione S-transferase  Glutathione S-transferase  Glutathione S-transferase  Glutathione S-transferase  Glutathione S-transferase  Glutathione S-transferase  Glutathione S-transferase  Glutathione S-transferase  Glutathione S-transferase  Glutathione S-transferase  Glutathione S-transferase  Glutathione S-transferase  Glutathione S-transferase  Glutathione S-transferase  Glutathione S-transferase  Glutathione S-transferase  Glutathione S-transferase  Glutathione S-transferase  Glutathione S-transferase  Glutathione S-transferase  Glutathione S-transferase  Glutathione S-transferase  Glutathione S-transferase  Glutathione S-transferase  Glutathione S-transferase  Glutathione S-transferase  Glutathione S-transferase  Glutathione S-transferase  Glutathione S-transferase  Glutathione S-transferase  Glutathione S-transferase  Glutathione S-transferase  Glutathione S-transferase  Glutathione S-transferase  Glutathione S-transferase  Glutathione S-transferase |

Table S2d. DEGs related to nitrogen metabolism, plant hormone signal transduction, Glutathione metabolism and transcription factors (TFs) between HN and HN+IBA

| Gene ID | log2 Ratio (HN+IBA/ HN) | Up-Down Regulation (HN /HN+IBA) | Gene annotation |
| --- | --- | --- | --- |
| \| CSA014068 \| \| --- \| \| MSTRG.11485 \| \| CSA018284 \| \| CSA020711 \| \| CSA006476 \| \| CSA006478 \| \| CSA027328 \| \| CSA023726 \| \| CSA003776 \| \| CSA022104 \| \| MSTRG.52360 \| \| MSTRG.15685 \| \| CSA017442 \| \| CSA029669 \| \| CSA024363 \| \| CSA004375 \| \| CSA028264 \| \| CSA018674 \| \| CSA011542 \| \| CSA023727 \| \| CSA023728 \| \| MSTRG.48424 \| \| CSA035763 \| \| CSA018345 \| \| CSA000188 \| \| CSA013711 \| \| CSA001095 \| \| CSA020581 \| \| CSA027936 \| \| CSA018435 \| \| CSA016141 \| \| CSA021194 \| \| CSA025184 \| \| CSA025185 \| \| CSA013252 \| \| MSTRG.33097 \| \| CSA015976 \| \| CSA015975 \| \| CSA015977 \| \| MSTRG.46245 \| \| CSA021429 \| \| CSA025674 \| \| CSA012908 \| \| CSA019392 \| \| MSTRG.8719 \| \| CSA033042 \| \| CSA029198 \| \| CSA015331 \| \| MSTRG.20513 \| \| MSTRG.51965 \| | \| -3.89699744 \| \| --- \| \| -3.89699744 \| \| -3.663169089 \| \| -3.369823522 \| \| -3.323195361 \| \| -3.323195361 \| \| -3.158855609 \| \| -3.14325343 \| \| -2.814522817 \| \| -2.788655855 \| \| -2.481092375 \| \| -2.479895262 \| \| -2.387960704 \| \| -2.3573915 \| \| -2.328108683 \| \| -2.30861005 \| \| -2.299487995 \| \| -2.26899516 \| \| -2.221797727 \| \| -2.212737668 \| \| -2.212737668 \| \| -2.197524831 \| \| -2.097945275 \| \| -2.07813255 \| \| -2.072283506 \| \| -2.03203183 \| \| -1.935786054 \| \| -1.911169255 \| \| -1.873910515 \| \| -1.790816983 \| \| -1.787375162 \| \| -1.63667042 \| \| -1.53702565 \| \| -1.53702565 \| \| -1.421796581 \| \| -1.322961286 \| \| -1.144724303 \| \| -1.144724303 \| \| -1.144724303 \| \| -1.101582686 \| \| 1.610426379 \| \| 2.091499399 \| \| 2.523170626 \| \| 2.558795528 \| \| 3.785352238 \| \| 4.330540278 \| \| 4.703224937 \| \| 5.463999031 \| \| 6.240903407 \| \| 7.018363326 \| | \| down \| \| --- \| \| down \| \| down \| \| down \| \| down \| \| down \| \| down \| \| down \| \| down \| \| down \| \| down \| \| down \| \| down \| \| down \| \| down \| \| down \| \| down \| \| down \| \| down \| \| down \| \| down \| \| down \| \| down \| \| down \| \| down \| \| down \| \| down \| \| down \| \| down \| \| down \| \| down \| \| down \| \| down \| \| down \| \| down \| \| down \| \| down \| \| down \| \| down \| \| down \| \| up \| \| up \| \| up \| \| up \| \| up \| \| up \| \| up \| \| up \| \| up \| \| up \| | \| Protein NRT1/ PTR FAMILY 1.2 \| \| --- \| \| Protein NRT1/ PTR FAMILY 1.2 \| \| Protein NRT1/ PTR FAMILY 2.6 \| \| Protein NRT1/ PTR FAMILY 4.5 \| \| Protein NRT1/ PTR FAMILY 5.1 \| \| Protein NRT1/ PTR FAMILY 5.1 \| \| Protein NRT1/ PTR FAMILY 3.1 \| \| Protein NRT1/ PTR FAMILY 5.5 \| \| Protein NRT1/ PTR FAMILY 4.5 \| \| Protein NRT1/ PTR FAMILY 5.5 \| \| Protein NRT1/ PTR FAMILY 1.2 \| \| Protein NRT1/ PTR FAMILY 1.2 \| \| Protein NRT1/ PTR FAMILY 6.3 \| \| Protein NRT1/ PTR FAMILY 2.11 \| \| Protein NRT1/ PTR FAMILY 3.1 \| \| Protein NRT1/ PTR FAMILY 3.1 \| \| Protein NRT1/ PTR FAMILY 3.1 \| \| Protein NRT1/ PTR FAMILY 4.6 \| \| Protein NRT1/ PTR FAMILY 3.1 \| \| Protein NRT1/ PTR FAMILY 5.5 \| \| Protein NRT1/ PTR FAMILY 5.5 \| \| Protein NRT1/ PTR FAMILY 1.2 \| \| Protein NRT1/ PTR FAMILY 5.2 \| \| Protein NRT1/ PTR FAMILY 8.1 \| \| Protein NRT1/ PTR FAMILY 3.1 \| \| Protein NRT1/ PTR FAMILY 7.3 \| \| Protein NRT1/ PTR FAMILY 5.2 \| \| Protein NRT1/ PTR FAMILY 4.6 \| \| Protein NRT1/ PTR FAMILY 5.6 \| \| Protein NRT1/ PTR FAMILY 3.1 \| \| Protein NRT1/ PTR FAMILY 3.1 \| \| Protein NRT1/ PTR FAMILY 5.9 \| \| Protein NRT1/ PTR FAMILY 2.3 \| \| Protein NRT1/ PTR FAMILY 2.5 \| \| Protein NRT1/ PTR FAMILY 5.2 \| \| Protein NRT1/ PTR FAMILY 1.2 \| \| Protein NRT1/ PTR FAMILY 6.1 \| \| Protein NRT1/ PTR FAMILY 6.1 \| \| Protein NRT1/ PTR FAMILY 6.1 \| \| Protein NRT1/ PTR FAMILY 2.2 \| \| Protein NRT1/ PTR FAMILY 8.3 \| \| Protein NRT1/ PTR FAMILY 2.13 \| \| Protein NRT1/ PTR FAMILY 4.3 \| \| Protein NRT1/ PTR FAMILY 5.5 \| \| Protein NRT1/ PTR FAMILY 5.16 \| \| Protein NRT1/ PTR FAMILY 5.6 \| \| Protein NRT1/ PTR FAMILY 7.3 \| \| Protein NRT1/ PTR FAMILY 7.3 \| \| Protein NRT1/ PTR FAMILY 1.2 \| \| Protein NRT1/ PTR FAMILY 1.2 \| |
| \| CSA004974 \| \| --- \| \| CSA004089 \| \| CSA010141 \| \| CSA019710 \| \| CSA025660 \| \| CSA021322 \| \| CSA011576 \| \| CSA029222 \| \| CSA012245 \| \| MSTRG.13397 \| \| MSTRG.45542 \| \| MSTRG.15556 \| \| CSA034099 \| \| CSA032238 \| \| CSA001824 \| \| CSA012931 \| \| CSA022603 \| \| CSA023080 \| \| CSA005481 \| | \| -1.968334294 \| \| --- \| \| -1.946639011 \| \| -1.93339478 \| \| -1.404961127 \| \| -1.27825437 \| \| -1.171371562 \| \| -1.03550097 \| \| 1.008502676 \| \| 1.97636245 \| \| 2.024114564 \| \| 2.024408448 \| \| 2.056479401 \| \| 2.918196842 \| \| 3.137450345 \| \| 3.544461237 \| \| 4.90022819 \| \| 4.93091404 \| \| 5.37769693 \| \| 5.92014361 \| | \| down \| \| --- \| \| down \| \| down \| \| down \| \| down \| \| down \| \| down \| \| up \| \| up \| \| up \| \| up \| \| up \| \| up \| \| up \| \| up \| \| up \| \| up \| \| up \| \| up \| | \| AUX/IAA family \| \| --- \| \| AUX/IAA family \| \| AUX/IAA family \| \| AUX/IAA family \| \| AUX/IAA family \| \| AUX/IAA family \| \| AUX/IAA family \| \| AUX/IAA family \| \| AUX/IAA family \| \| AUX/IAA family \| \| AUX/IAA family \| \| AUX/IAA family \| \| AUX/IAA family \| \| AUX/IAA family \| \| AUX/IAA family \| \| AUX/IAA family \| \| AUX/IAA family \| \| AUX/IAA family \| \| AUX/IAA family \| |
| \| CSA013605 \| \| --- \| \| CSA035666 \| \| CSA021293 \| \| CSA018958 \| \| CSA007332 \| \| CSA004727 \| \| CSA032194 \| \| CSA013122 \| \| CSA033915 \| \| CSA013292 \| | \| -3.650101443 \| \| --- \| \| -3.460455688 \| \| -2.613739362 \| \| -1.138015239 \| \| 1.069208017 \| \| 1.328945116 \| \| 1.386460003 \| \| 5.299503538 \| \| 9.070007527 \| \| 9.454678024 \| | \| down \| \| --- \| \| down \| \| down \| \| down \| \| up \| \| up \| \| up \| \| up \| \| up \| \| up \| | \| GH3 auxin-responsive promoter \| \| --- \| \| GH3 auxin-responsive promoter \| \| GH3 auxin-responsive promoter \| \| GH3 auxin-responsive promoter \| \| GH3 auxin-responsive promoter \| \| GH3 auxin-responsive promoter \| \| GH3 auxin-responsive promoter \| \| GH3 auxin-responsive promoter \| \| GH3 auxin-responsive promoter \| \| GH3 auxin-responsive promoter \| |
| \| CSA000131 \| \| --- \| \| MSTRG.10841 \| \| MSTRG.51720 \| \| MSTRG.45862 \| \| CSA032051 \| \| CSA016398 \| \| CSA022556 \| \| CSA022159 \| \| CSA009028 \| \| CSA018325 \| \| CSA018326 \| \| MSTRG.29213 \| \| MSTRG.52319 \| \| CSA027875 \| \| CSA014568 \| \| CSA014569 \| \| CSA032833 \| \| MSTRG.41821 \| \| CSA031833 \| \| MSTRG.16244 \| \| CSA004940 \| \| MSTRG.3898 \| \| MSTRG.38013 \| \| MSTRG.12251 \| \| MSTRG.20996 \| \| CSA003014 \| | \| -5.707477006 \| \| --- \| \| -5.144195864 \| \| -4.520370149 \| \| -3.112084733 \| \| -2.423635005 \| \| -2.417566463 \| \| -2.326456631 \| \| -1.877438398 \| \| -1.624042425 \| \| -1.377251473 \| \| -1.377251473 \| \| -1.064921087 \| \| -1.025370192 \| \| -1.013980339 \| \| 1.008793837 \| \| 1.008793837 \| \| 1.215422818 \| \| 1.251757886 \| \| 1.638359174 \| \| 1.773751894 \| \| 2.024895425 \| \| 2.57105226 \| \| 3.158387793 \| \| 4.0979029 \| \| 5.684373354 \| \| 6.120953727 \| | \| down \| \| --- \| \| down \| \| down \| \| down \| \| down \| \| down \| \| down \| \| down \| \| down \| \| down \| \| down \| \| down \| \| down \| \| down \| \| up \| \| up \| \| up \| \| up \| \| up \| \| up \| \| up \| \| up \| \| up \| \| up \| \| up \| \| up \| | \| MADS-box protein \| \| --- \| \| MADS-box protein \| \| MADS-box protein \| \| MADS-box protein \| \| MADS-box protein \| \| MADS-box protein \| \| MADS-box protein \| \| MADS-box protein \| \| MADS-box protein \| \| MADS-box protein \| \| MADS-box protein \| \| MADS-box protein \| \| MADS-box protein \| \| MADS-box protein \| \| MADS-box protein \| \| MADS-box protein \| \| MADS-box protein \| \| MADS-box protein \| \| MADS-box protein \| \| MADS-box protein \| \| MADS-box protein \| \| MADS-box protein \| \| MADS-box protein \| \| MADS-box protein \| \| MADS-box protein \| \| MADS-box protein \| |
| \| CSA009011 \| \| --- \| \| CSA007495 \| \| MSTRG.38842 \| \| CSA009570 \| \| CSA010605 \| \| CSA025190 \| \| CSA023709 \| \| CSA001761 \| \| CSA008907 \| \| CSA017287 \| \| MSTRG.35982 \| \| CSA021806 \| \| CSA023222 \| \| CSA023223 \| \| CSA017619 \| \| CSA015968 \| \| CSA013362 \| \| MSTRG.24765 \| \| CSA026809 \| \| CSA010950 \| \| CSA004463 \| \| CSA017618 \| \| CSA017617 \| \| MSTRG.23150 \| \| MSTRG.30887 \| \| CSA023607 \| \| CSA017488 \| \| CSA013925 \| \| MSTRG.32666 \| \| CSA023606 \| \| CSA010932 \| \| CSA010933 \| \| CSA009919 \| \| MSTRG.3755 \| \| MSTRG.41801 \| \| MSTRG.45308 \| \| MSTRG.36581 \| \| MSTRG.2854 \| \| MSTRG.41840 \| \| CSA003142 \| \| MSTRG.2856 \| \| CSA004457 \| \| CSA025566 \| | \| -2.881036246 \| \| --- \| \| -2.619761702 \| \| -1.909417354 \| \| -1.867402265 \| \| -1.680411663 \| \| -1.642083183 \| \| -1.420969377 \| \| -1.404373359 \| \| 1.10410687 \| \| 1.108903882 \| \| 1.113478531 \| \| 1.177023941 \| \| 1.22952499 \| \| 1.22952499 \| \| 1.628379124 \| \| 1.849351628 \| \| 1.911259731 \| \| 1.997290371 \| \| 2.161937632 \| \| 2.202284375 \| \| 2.205366471 \| \| 2.278211693 \| \| 2.278211693 \| \| 2.581186974 \| \| 2.749430604 \| \| 3.580993964 \| \| 4.507055467 \| \| 4.521041069 \| \| 4.616480582 \| \| 4.683055824 \| \| 4.700104241 \| \| 4.700104241 \| \| 5.051571314 \| \| 5.395568351 \| \| 5.778957204 \| \| 5.78875729 \| \| 5.931595947 \| \| 6.214056132 \| \| 6.264369226 \| \| 6.420051501 \| \| 6.513403057 \| \| 6.525655278 \| \| 7.199499949 \| | \| down \| \| --- \| \| down \| \| down \| \| down \| \| down \| \| down \| \| down \| \| down \| \| up \| \| up \| \| up \| \| up \| \| up \| \| up \| \| up \| \| up \| \| up \| \| up \| \| up \| \| up \| \| up \| \| up \| \| up \| \| up \| \| up \| \| up \| \| up \| \| up \| \| up \| \| up \| \| up \| \| up \| \| up \| \| up \| \| up \| \| up \| \| up \| \| up \| \| up \| \| up \| \| up \| \| up \| \| up \| | \| NAC domain-containing protein \| \| --- \| \| NAC domain-containing protein \| \| NAC domain-containing protein \| \| NAC domain-containing protein \| \| NAC domain-containing protein \| \| NAC domain-containing protein \| \| NAC domain-containing protein \| \| NAC domain-containing protein \| \| NAC domain-containing protein \| \| NAC domain-containing protein \| \| NAC domain-containing protein \| \| NAC domain-containing protein \| \| NAC domain-containing protein \| \| NAC domain-containing protein \| \| NAC domain-containing protein \| \| NAC domain-containing protein \| \| NAC domain-containing protein \| \| NAC domain-containing protein \| \| NAC domain-containing protein \| \| NAC domain-containing protein \| \| NAC domain-containing protein \| \| NAC domain-containing protein \| \| NAC domain-containing protein \| \| NAC domain-containing protein \| \| NAC domain-containing protein \| \| NAC domain-containing protein \| \| NAC domain-containing protein \| \| NAC domain-containing protein \| \| NAC domain-containing protein \| \| NAC domain-containing protein \| \| NAC domain-containing protein \| \| NAC domain-containing protein \| \| NAC domain-containing protein \| \| NAC domain-containing protein \| \| NAC domain-containing protein \| \| NAC domain-containing protein \| \| NAC domain-containing protein \| \| NAC domain-containing protein \| \| NAC domain-containing protein \| \| NAC domain-containing protein \| \| NAC domain-containing protein \| \| NAC domain-containing protein \| \| NAC domain-containing protein \| |
| \| CSA013924 \| \| --- \| \| CSA016850 \| \| CSA023014 \| \| CSA014257 \| \| MSTRG.27188 \| \| MSTRG.47422 \| \| CSA019809 \| \| CSA012171 \| \| MSTRG.47297 \| \| CSA027168 \| \| CSA007816 \| \| CSA007248 \| \| CSA030472 \| \| CSA008877 \| \| CSA017015 \| \| CSA027870 \| \| CSA027604 \| \| CSA013458 \| \| CSA026928 \| \| CSA033166 \| \| CSA022209 \| \| CSA024123 \| \| CSA030064 \| \| CSA003378 \| \| CSA003379 \| \| CSA005706 \| \| CSA005707 \| \| MSTRG.13572 \| \| CSA004281 \| \| CSA023591 \| \| CSA001952 \| \| CSA003106 \| \| MSTRG.526 \| \| MSTRG.28367 \| \| CSA008627 \| \| CSA002710 \| \| MSTRG.50473 \| \| CSA030708 \| \| MSTRG.42422 \| \| CSA036012 \| \| CSA009535 \| \| MSTRG.12649 \| \| MSTRG.26226 \| \| CSA016849 \| \| CSA019504 \| \| CSA029390 \| \| CSA021419 \| | \| -4.945935163 \| \| --- \| \| -3.792017864 \| \| -3.382700779 \| \| -3.344274403 \| \| -2.419355699 \| \| -2.282749294 \| \| -2.125430485 \| \| -1.854057154 \| \| -1.667777929 \| \| -1.631803759 \| \| -1.61752808 \| \| -1.580268935 \| \| -1.570157036 \| \| -1.101793179 \| \| -1.053272797 \| \| -1.011997064 \| \| 1.00354218 \| \| 1.02969853 \| \| 1.074272952 \| \| 1.135542367 \| \| 1.169316462 \| \| 1.29397909 \| \| 1.302126299 \| \| 1.343050323 \| \| 1.343050323 \| \| 1.366832328 \| \| 1.366832328 \| \| 1.434825498 \| \| 1.532278769 \| \| 1.64576023 \| \| 1.696750174 \| \| 1.707931619 \| \| 1.751114948 \| \| 1.868545097 \| \| 2.171263155 \| \| 2.456380817 \| \| 2.615384857 \| \| 2.633604588 \| \| 2.689284901 \| \| 2.901363802 \| \| 3.156453119 \| \| 3.209784181 \| \| 3.242051064 \| \| 3.530648138 \| \| 3.766176906 \| \| 5.035083505 \| \| 5.379413306 \| | \| down \| \| --- \| \| down \| \| down \| \| down \| \| down \| \| down \| \| down \| \| down \| \| down \| \| down \| \| down \| \| down \| \| down \| \| down \| \| down \| \| down \| \| up \| \| up \| \| up \| \| up \| \| up \| \| up \| \| up \| \| up \| \| up \| \| up \| \| up \| \| up \| \| up \| \| up \| \| up \| \| up \| \| up \| \| up \| \| up \| \| up \| \| up \| \| up \| \| up \| \| up \| \| up \| \| up \| \| up \| \| up \| \| up \| \| up \| \| up \| | \| WRKY transcription factor \| \| --- \| \| WRKY transcription factor \| \| WRKY transcription factor \| \| WRKY transcription factor \| \| WRKY transcription factor \| \| WRKY transcription factor \| \| WRKY transcription factor \| \| WRKY transcription factor \| \| WRKY transcription factor \| \| WRKY transcription factor \| \| WRKY transcription factor \| \| WRKY transcription factor \| \| WRKY transcription factor \| \| WRKY transcription factor \| \| WRKY transcription factor \| \| WRKY transcription factor \| \| WRKY transcription factor \| \| WRKY transcription factor \| \| WRKY transcription factor \| \| WRKY transcription factor \| \| WRKY transcription factor \| \| WRKY transcription factor \| \| WRKY transcription factor \| \| WRKY transcription factor \| \| WRKY transcription factor \| \| WRKY transcription factor \| \| WRKY transcription factor \| \| WRKY transcription factor \| \| WRKY transcription factor \| \| WRKY transcription factor \| \| WRKY transcription factor \| \| WRKY transcription factor \| \| WRKY transcription factor \| \| WRKY transcription factor \| \| WRKY transcription factor \| \| WRKY transcription factor \| \| WRKY transcription factor \| \| WRKY transcription factor \| \| WRKY transcription factor \| \| WRKY transcription factor \| \| WRKY transcription factor \| \| WRKY transcription factor \| \| WRKY transcription factor \| \| WRKY transcription factor \| \| WRKY transcription factor \| \| WRKY transcription factor \| \| WRKY transcription factor \| |
| \| CSA013844 \| \| --- \| \| CSA032576 \| \| CSA032575 \| \| MSTRG.31742 \| \| MSTRG.45200 \| | \| 1.088056232 \| \| --- \| \| 2.019235488 \| \| 2.019235488 \| \| 3.053747829 \| \| 3.656298985 \| | \| up \| \| --- \| \| up \| \| up \| \| up \| \| up \| | \| Glutathione reductase \| \| --- \| \| Glutathione reductase \| \| Glutathione reductase \| \| Glutathione reductase \| \| Glutathione reductase \| |
